# Advanced Nickel-Based Gas Diffusion Anode for Zero-Gap Anion-Exchange Membrane Water Electrolyzers

**DOI:** 10.1021/acsami.5c01272

**Published:** 2025-05-23

**Authors:** Irina V. Pushkareva, Zhixing Wu, Xianjie Liu, Maksim A. Solovyev, Sergey I. Butrim, Margarita V. Kozlova, Tatiana L. Kulova, Reverant Crispin, Emma M. Björk, Dmitri G. Bessarabov, Mikhail Vagin, Artem S. Pushkarev

**Affiliations:** † HySA Infrastructure Center of Competence, Faculty of Engineering, 56405 North-West University, Private Bag X6001, Potchefstroom Campus, 2531 Potchefstroom, South Africa; ‡ Laboratory of Organic Electronics, Department of Science and Technology (ITN), 4566 Linköping University, 60174 Norrköping, Sweden; § National Research Center “Kurchatov Institute” 1, Kurchatov Sq., Moscow 123182, Russia; ∥ National Research University “Moscow Power Engineering Institute”, 14, Krasnokazarmennaya Str., 111250 Moscow, Russia; ⊥ Frumkin Institute of Physical Chemistry and Electrochemistry, Russian Academy of Sciences, 31-4 Leninskii Ave., 119071 Moscow, Russia; # Nanostructured Materials, Department of Physics, Chemistry and Biology (IFM), Linköping University, SE58183 Linköping, Sweden; ∇ Wallenberg Initiative Materials Science for Sustainability, Department of Science and Technology, Linköping University, 60174 Norrköping, Sweden; ○ Wallenberg Wood Science Center, Linköping University, 60174 Norrköping, Sweden

**Keywords:** water electrolysis, anion-exchange membrane, membrane electrode assembly, nickel foam, microporous layer, mesoporous nickel(II) oxide, electrochemical impedance spectroscopy

## Abstract

Electrolytic hydrogen production using abundant water and renewable electricity is a key step toward achieving a carbon-neutral economy. Anion-exchange membrane water electrolyzers (AEMWE) present an opportunity to enhance sustainability and reduce the costs of green hydrogen technology. This study focuses on reducing electrical losses in the AEMWE by designing an improved anode catalyst layer. The approach involves modifying nickel foam by using a microporous nickel ink. This modification not only smooths the nickel foam to prevent membrane punctures during compression assembly but also enhances the utilization of the mesoporous NiO (mesoNiO) catalyst in the anode process, namely, the oxygen evolution reaction (OER). The anode leverages a mechanism where both the mesoNiO catalyst and the nickel powder layer participate in the OER, hosting a NiOOH intermediate formed through surface oxidation. By optimization of the mass loading, the design achieves a balance between smooth membrane–electrode contact, reduced kinetic losses during the OER, and efficient ionic transport. As a result, the optimized AEMWE reaches a competitive current density of 2.6 A cm^–2^ at a cell voltage of 2 V, comparable to the performance of state-of-the-art proton-exchange membrane water electrolyzers. These findings highlight that fluorocarbon membrane-free, zero-gap water electrolyzers with platinum-free anodes can deliver significant advancements in green hydrogen technology. This promising performance encourages further research toward catalyst-free water electrolyzers as the next step in sustainable hydrogen production.

## Graphical abstract

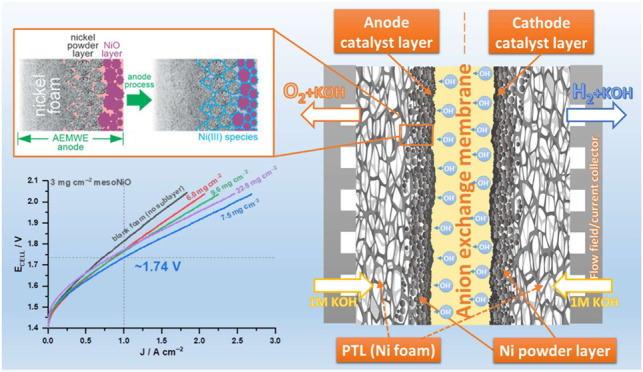

## 1 Introduction

In the chemical industry, the direct use of electricity from renewable sources contributes to some of the ‘Sustainable Development Goals’ since it bypasses greenhouse gas emissions.^1^ Hydrogen production via water electrolysis, using electricity from renewables—so-called green hydrogen—plays a key role in the decarbonization of a few sectors that produce most of the greenhouse gas, including energy,^2^ steel,^3^ and ammonia production.^4^ However, the high cost of green hydrogen technology limits its current share of global hydrogen production to be as low as 5%.

Pertaining to the key factors required for fast-response and loss-efficient water electrolyzers, it is the high ionic conductivity, in combination with a capability to block the cross-contamination of gaseous products, namely, hydrogen and oxygen, that define the properties of the membrane sandwiched between an anode and a cathode. In the construction of WE, it is the transport of a proton or hydroxide anion, respectively, through the membrane that determines the suitable use of a proton-exchange membrane (PEM) or an anion-exchange membrane (AEM).^5–8^ High proton conductivity combined with excellent barrier properties has been achieved with sulfonated fluoropolymers such as Nafion, contributing to their wide use in PEM water electrolyzers (PEMWE).

However, disadvantages of the family of PEMs include the following three issues: their high cost, the fact that the manufacturing byproducts are classified as undesirable per- and polyfluoroalkyl materials^9^—persistent organic pollutants posing health and environmental risks—and the requirement for noble metals as catalysts for the anode process of PEMWE, namely, the oxygen evolution reaction (OER; H_2_O→O_2_ + 4H^+^ + 4e^–^).

The development of water electrolyzers based on AEMs contributes to sustainable green hydrogen technology. This is largely due to the fluoride-free chemistry of an AEM and the possibility of using cost-efficient anode catalysts based on transition metals.^10–14^ Recent life cycle analysis reports reveal that AEMWE outperforms both PEMWE and traditional alkaline water electrolysis in terms of climate and environmental impact.^15,16^ Even better results are expected soon due to its currently lower technology readiness level and potential for design improvements free from critical raw materials.

In this regard, NiO was recently suggested as an active and cost-efficient OER catalyst.^17–19^ The hydrogen production process at the cathode of an AEM water electrolyzer (AEMWE) is as follows

1 $$2H_{2}O+2e^{-}\to H_{2}+2OH^{-}$$

while the OER, auxiliary process on an AEMWE anode, is as follows

2 $$4OH^{-}\to O_{2}+2H_{2}O+4e^{-}$$

The complex multistep mechanism of the direct OER (reaction 2) and the reverse process (hydrogen evolution reaction, reaction 1) defines the sluggish kinetics of these processes hosted on electrodes. This results in the limitation of devices based on electrodes for direct oxygen generation and use, e.g., water electrolyzers and fuel cells. The role of the catalyst here is to afford vigorous oxygen-associated processes, which will result in a reduction of the electrical energy losses in green hydrogen technologies.

To minimize the ohmic losses in electrolyzers, a compressed sandwich between the membrane and the electrodes, namely, the anode on one side of the membrane and the cathode on the other side, is created, giving the zero-gap membrane electrode assembly (MEA). Porous electrode substrates are used in an assembly to ensure the fast transport of reagents and products through the large pores of the electrodes,^20^ so-called porous transport layers. Various porous materials are utilized for this purpose, including carbon felt,^21^ stainless-steel felt,^22,23^ and nickel foam.^24,25^ However, the direct contact of porous substrates with the membrane in an electrolyzer assembly is unfavorable due to the low number of contact points^12^ and the high risk of membrane puncture.^26^ Moreover, the direct deposition of the catalyst required to mitigate the electrical losses due to the slow kinetics of electrode processes, namely, reactions 1 and 2, on the porous electrode substrate before the electrolyzer assembly is also unfavorable. This is due to the significant loss of catalyst in the bulk of the porous substrate, making it inaccessible for the electrode process,^27–29^ the high increase in contact resistance between the electrode and membrane,^30^ and the possibility of layer delamination.^31^

Therefore, the minimization of electrical losses on water electrolyzers requires the engineering of a suitable layer between the membrane and the porous electrode substrate to create a smooth contact and to provide catalytic functionality. The objective was therefore to create a catalyst layer either on a porous substrate or on a membrane— a so-called catalyst-coated substrate (CCS)^24^ or catalyst-coated membrane,^32^ respectively.

We were inspired by the fabrication of electrodes for PEM fuel cells,^33^ where the interfaces between the membrane and the porous electrode substrate are created by the formation of an additional microporous layer, a so-called sublayer.^34–37^ Such a ‘smoothening layer’ improves both the contact resistance and the accessibility of the catalyst, while the transport of products and reagents across it should remain unaffected. This illustrates the need for the optimization of the smoothening layer.^36,38^ Such optimization of a sintered titanium sublayer has enabled PEMWE operation under extreme conditions (current density 6 A cm^–2^, 90 °C, and 90 bar pressure).^37^

In the present study, we investigated the effect of a nickel powder layer deposited on nickel foam to minimize the electrical losses of AEMWE arising from membrane–electrode contact and the slow kinetics of the anode process. Optimization of the deposited layers enabled us to achieve a balance between the smoothened contact resistance, enhanced OER catalysis, and ionic transport across the layer. Concurrently, for the mesoNiO catalyst, the nickel powder layer exhibited involvement in the OER catalysis. The obtained characteristics of AEMWE are similar to the characteristics of PEMWE, thus illustrating a contribution toward the sustainability of green hydrogen technology.

## 2 Experimental Section

### 2.1 Materials

Nickel­(II) chloride hexahydrate (≥98%), ammonium hydroxide solution (28–30%), sodium dodecyl sulfate (SDS; ≥ 99%), isopropyl alcohol, potassium hydroxide, Nafion perfluorinated resin solution (5 wt %), and potassium hydroxide were purchased from Sigma-Aldrich. Milli-Q water was used in all experiments. The following materials were used for membrane electrode assemblies: Sustainion X37–50 (Dioxide Materials), Pt/C catalyst PM40 (Prometheus R&D, LLC, Russia), Ni foam (OhmLiberScience, Russia), and nickel powder (GOST 9722–97, Ruschim, Russia).

### 2.2 Preparation of NiO

MesoNiO was prepared by a previously developed hydrothermal synthesis method.^39^ Typically, nickel­(II) chloride and SDS (molar ratio 1:2; 1 wt % SDS) were dissolved in Milli-Q water at room temperature under vigorous stirring, affording a transparent solution. Ammonia hydroxide was added dropwise to the solution until pH 10 was reached. After being stirred for 5 h, the mixture was transferred to a polytetrafluoroethylene bottle and placed in an oven for hydrothermal treatment at 100 °C for 2 days. The collected green precursor was washed with ethanol and water several times. The precursor was calcined in a muffle furnace under an ambient atmosphere at 400 °C for 4 h (temperature ramp 10 °C min^–1^).

### 2.3 Physicochemical Characterization

The mesoNiO crystal structure was determined by X-ray diffraction (XRD) using a PANalytical X’Pert Pro X-ray diffractometer (Malvern Panalytical, The Netherlands) with Cu Kα radiation (λ = 0.15406 nm). Physisorption measurements were carried out on a Micromeritics ASAP 2020 analyzer (Micromeritics Instrument Corporation) at −196 °C using nitrogen as an absorbent. Brunauer–Emmett–Teller (BET) and Barrett–Joyner–Halenda methods were used to calculate the specific surface area and pore volume. The desorption isotherm was used to calculate pore size distributions. The surface state of materials was characterized by X-ray photoelectron spectroscopy (XPS) and X-ray absorption spectroscopy (XAS). XPS measurements were performed using a Scienta ESCA 200 system (Scienta Omicron, Germany) under a base pressure of 2E–10 mbar, equipped with an SES 200 electron analyzer and a monochromatic Al Kα X-ray source (hν 1486.6 eV). All spectra were collected at room temperature with normal emission. Prior to the measurements, the spectrometer was calibrated using a sputter-cleaned Au film: Fermi level 0 eV and Au 4f7/2 peak at 84.0 eV, with full width half-maximum 0.65 eV. The Ni L2, 3 absorption edge XAS spectra were collected in total electron mode at FinEstBeAMS beamline in MAX IV Lund with an energy resolution of about 0.1 eV. All XAS spectra were normalized by the incident flux, as measured by the current from a clean gold mesh along the monochromatic beam path before the incident on the sample.

### 2.4 Half-Cell Electrochemical Measurements

Evaluation of the OER activity of nickel powder and synthesized mesoNiO was carried out in an alkaline medium (oxygen-saturated 1 M KOH) using cyclic voltammetry (CV), controlled by an SP-200 (BioLogic, France) potentiostat on a rotating disk electrode setup (Pine Research Instrumentations). A glassy carbon electrode (GCE) (with a 5 mm diameter), graphite rod, and Hg/HgO were used as the rotating disk working electrode (rotation speed 1600 rpm) and the counter and reference electrodes, respectively (Scheme 1A,B). The ohmic drop compensation was carried out manually. The scale of applied potentials was transformed by the Nernst equation with respect to the reversible hydrogen electrode (RHE).

**Scheme 1:**
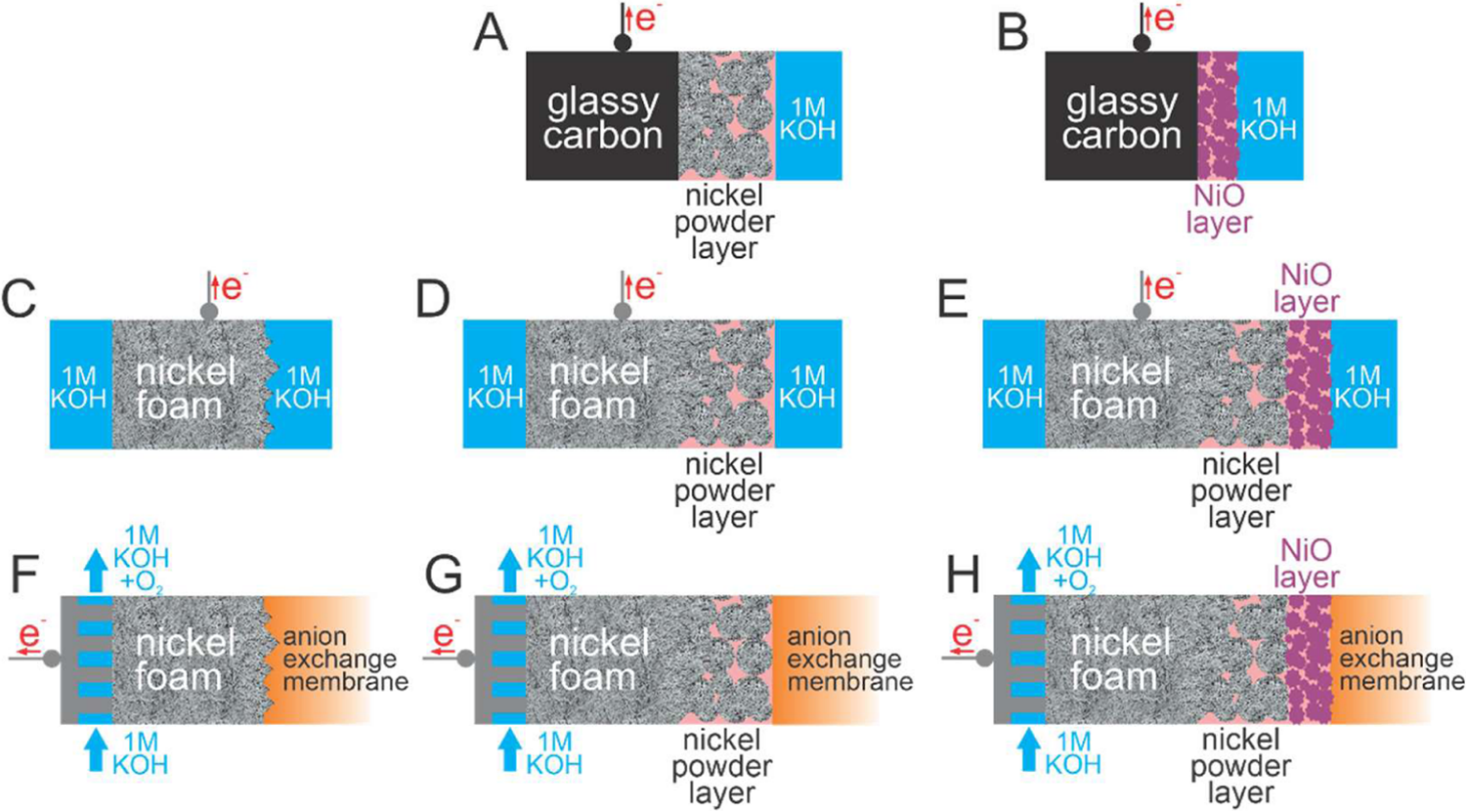
Electrode Systems^a^

a (A, B): modified glassy carbon electrodes (three-electrode cell); (C–E): ‘flag’ electrodes (three-electrode cell); and (F–H): AEMWE anodes.

To prepare a homogeneous nickel powder and mesoNiO ink of 10 mg mL^–1^ concentration, 10 mg of catalyst was dispersed in a solution containing 980 μL of ethanol and 20 μL of 5 wt % Nafion solution, with sonication. A small quantity of the ink was drop-coated onto a GCE, followed by drying in an oven at 60 °C for 30 min.

The blank and modified nickel foam samples (rectangular “flags”, ∼5 × 10 mm^2^) were cut and hooked on a platinum hook. Each of the individual hooked samples was fully immersed in 1 M KOH and then used as a working electrode in a three-electrode cell filled with 1 M KOH (Scheme 1C–E).

### 2.5 AEMWE Measurements

The assembly of AEMWE was carried out by means of the CCS approach, using a commercially available AEM (Sustainion X37–50). Samples of nickel foam (size 2.7 × 2.7 cm^2^, initial thickness 900 μm, porosity 90–95%, 110 PPI) were compressed to a final thickness of 300 μm. The nickel ink was prepared by dispersion of nickel powder (particle size ∼ 3–20 μm, Figure S2) in 5 wt % Nafion solution. The dispersions of mesoNiO and platinum on carbon (PM40)^40^ in 5 wt % Nafion solution were used as anode and cathode catalytic inks, respectively.

The cathode was prepared by spray-coating cathode catalytic ink (mass loading 0.8 mg cm^–2^) onto one side of the compressed nickel foam.^41^ The anode was fabricated by brush-coating nickel ink onto one side of the compressed nickel foam (mass loading 6–23 mg cm^–2^). To deposit the anode catalyst, the anode catalyst ink was spray coated onto an anode (mass loading 0–3 mg cm^–2^) (Scheme 1F–H).

Before cell assembly, the membrane was activated in 1 M KOH for 24 h at room temperature (according to the manufacturer’s recommendations). The membrane was sandwiched between the anode and cathode (with the modified sides facing the membrane) and fixed in the electrolyzer, made of two stainless-steel compartments with engraved flow fields (parallel channels).

A test bench described was used for AEMWE performance evaluation (Figure S1). Both anode and cathode compartments were fed with 1 M KOH (flow rate of 3 mL min^–1^) using the two-channel peristaltic pump. Anode and cathode output solutions were mixed in a sealed glassy tank with stirring to minimize carbonate buildup and ensure a constant electrolyte concentration. To avoid the loss of the electrolyte by released gases, the anode and cathode outlet flows were equipped with gas separators. The electrolyzer was operated at atmospheric pressure on both sides. No stainless-steel components/parts of the test bench were in contact with the KOH solution. Preconditioning of the electrolyzer was carried out by holding the device at 60 °C for 2 h under the different voltage steps consequently applied (at 1.7 and 2.0 V for 0.5 h each step and at 2.2 V for 1 h) until reproducible polarization behavior was obtained.

The performance of AEMWE was evaluated using an SI 1280 potentiostat (Ametek Inc.) equipped with a power booster 12 V/20 A (Ametek Inc.). Polarization curves were measured in potentiodynamic mode (1.4–2.05 V) at a sweep rate of 1 mV s^–1^.

Electrochemical impedance spectroscopy (EIS) data were collected after polarization curve measurements under potentiostatic control in the frequency range between 20 kHz and 100 mHz, with 10 mV amplitude. Prior to collection of the EIS data, the electrolyzer was conditioned at the applied potentials for 300 s. Processing of EIS data was performed using RelaxIs 3.0.2.19 software (rhd instruments GmbH & Co. KG, Germany). An example of Kramers–Kronig (KK) validity testing is provided in the Supporting Information (SI) (Figure S10). The high accuracy of the fitting is proven by the rather low values of the reduced chi-square factor: 5–50 × 10^–5^.

Scanning electron microscopy (SEM) analysis of the fabricated films was performed by using a Helios NanoLab 600i instrument (FEI).

## 3 Results and Discussion

### 3.1 Surface Area of Anodes

The deposition of a nickel powder layer increases the smoothness of the nickel foam (Figure 1), which is beneficial in terms of reducing the risk of membrane puncture by compression at the assembly or due to membrane swelling during electrolyzer operation.^42^ The blank nickel foam shows deep channels between neighboring ribs (size 50–100 μm) (Figure 1A). The attachment of nickel particles (Figure S2) is visible upon modification with low powder loading (6 mg cm^–2^, Figure 1B). A further increase in the mass loading of modification leads to significant filling of the channels and the attachment of nickel particles inside the foam. The high loading of the nickel foam by nickel powder particles (22.8 mg cm^–2^) almost blocks the large channels (Figure 1E).

**Figure 1:**
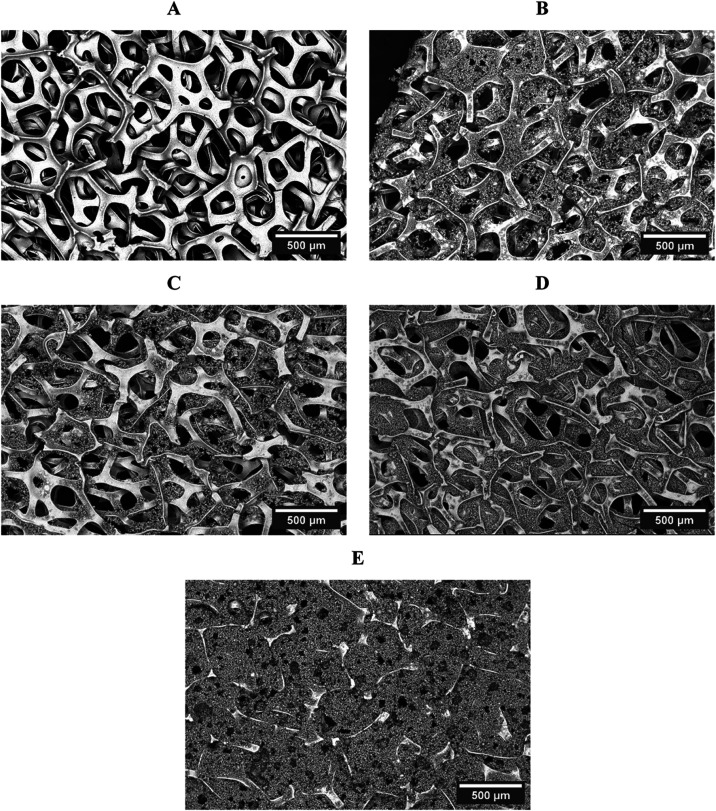
SEM images of pristine (A) and nickel powder-loaded nickel foams ((B)**:** 6 mg cm^–2^, (C): 7.5 mg cm^–2^, (D): 9.6 mg cm^–2^ and (E): 22.8 mg cm^–2^).

Direct contact between a metal electrode and electrolyte solution results in the formation of an electrical double layer at the interface. In such a situation, the capacitance of the electrode is the quantitative characteristic of the surface area of the metal available for the establishment of the electrical double layer, namely, the electrochemically available surface area (EASA).

We then investigated the evolution of EASA of nickel foam upon modification with nickel powder. Samples of blank and powder-modified nickel foams were used as working electrodes in a three-electrode cell filled with 1 M KOH (Scheme 1C,D). The currents recorded in CV experiments in the range of potentials far from faradaic processes (e.g., the OER, oxygen reduction reaction, and Ni^2+^/Ni^3+^ redox process) represent the capacitive behavior independent of the applied potentials. Normalization of the recorded currents on the scan rate and the total mass of the evaluated electrode yields the dependence of the mass-normalized electrode capacitance on the applied potential in CV experiments (Figure 2A). An increase in the foam loading with the nickel powder led to a linear increase in the mass-normalized electrode capacitance (e.g., at 0.95 V (RHE), Figure 2B), manifesting the expansion of the EASA. In agreement with CV results, the increase in nickel foam loading due to the nickel powder shows an increase in the total capacitance of the electrode as estimated from EIS measurements at a constant potential of 0.95 V (RHE) (Supporting Note 1, Figure S3B). Assuming the identical surface state of nickel foam and nickel powder particles on pristine samples, one can conclude that modification of the foam with metal ink increases its EASA. Specifically, a more than 3-fold increase in the loading (from 6 to 22.4 mg cm^–2^) resulted in a 2-fold increase in EASA, as evaluated by both CV and EIS.

**Figure 2:**
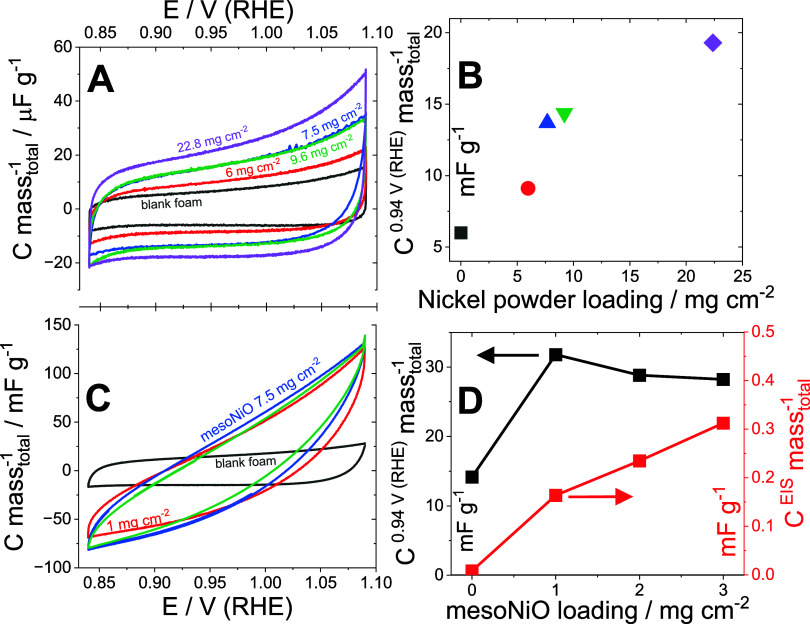
Evolution of EASA due to the modification of compressed nickel foam. (A): cyclic voltammograms obtained on compressed nickel foam modified by nickel powder of different loadings (three-electrode cell filled with 1 M KOH, Scheme 1D) normalized by the scan rate and by total electrode mass; (B): dependence of the mass-normalized capacitance on the nickel powder loading, estimated from CV (three-electrode cell, scan rate 50 mV s^–1^), currents at 0.94 V (RHE) on the nickel powder loading; (C): cyclic voltammograms obtained on compressed nickel foam modified by mesoNiO powder (constant loading of nickel powder (7.5 mg cm^–2^) under the mesoNiO layer; three-electrode cell filled with 1 M KOH, Scheme 1E); (D): dependences of mass-normalized capacitances estimated from CV (three-electrode cell, scan rate 50 mV s^–1^), current 0.94 V (RHE) and from EIS (0.94 V (RHE), amplitude 10 mV) on the mesoNiO (constant loading of nickel powder (7.5 mg cm^–2^) under the mesoNiO layer).

Dynamic behavior of EASA is evident with an increase in the voltammetry scan rate (Figure S4A). In contrast to a blank foam, the modified foam of any nickel powder loading showed poor retention of capacitance with an increase in the voltammetry scan rate (Figure S4B). This is due to the limitation of ionic transport caused by the relaxation of the electrical double layer formed on the porous metal surface. On the contrary, the blank foam showed a high retention of capacitance. Consistently with voltammetry, increased frequency in the EIS measurements led to a decrease in total capacitance (Figure S3C). An increased nickel powder loading resulted in a higher sensitivity to an increase in the frequency. These observations provide evidence of the limitation of ionic transport on a modified foam, which could be due to both the high porosity of the metal electrodes and the limited ionic transport offered by the Nafion binder.

We used a three-electrode cell to trace the evolution of EASA upon the deposition of mesoNiO on top of nickel foam modified with nickel powder (Scheme 1E). The selected conditions for mesoNiO synthesis were optimized previously to achieve mesoporosity and high OER currents.^39^ Characterization of the synthesized mesoNiO confirmed the presence of mesoporosity (Supporting Note 2), which manifested the high surface area catalyst of the OER. The poor electrical conductivity in comparison with nickel metal and the high surface area of mesoporous semiconductor mesoNiO resulted in the appearance of a resistive contribution in the CV response (Figure 2C). This led to a nonlinear dependence of total capacitance, estimated from voltammetry data (Figure 2D), on the mass loading of mesoNiO. This then motivated us to use EIS at a constant potential of 0.95 V (RHE) to estimate the EASA-associated capacitance (Supporting Note 3). An increase in the mesoNiO loading led to a linear increase in the electrode capacitance normalized by the total electrode mass, which implies a linear increase in EASA.

### 3.2 Faradaic Process on the Anode

To achieve the oxygen process limitation on AEMWE, we ensured that the cathode was fast enough not to be limiting. Specifically, a high-loading platinum catalyst on carbon was used on the cathode, producing hydrogen via the hydrogen evolution reaction (HER) (reaction 1). Such a configuration assures the assignment of the observed changes in the performance to the oxygen-evolving anode of the AEMWE.

The archetypical polarization curves obtained on AEMWE (Figure 3A,C) assembled with anodes of different loadings of nickel powder and mesoNiO catalyst (Scheme 1H), respectively, and showed an achievement of high current densities at certain cell voltages. The standard current density of 1 A cm^–2^ was achieved at cell voltages of 1.74–1.77 and 1.81 V on AEMWE with anodes based on nickel powder-loaded and pristine nickel foams, respectively. The characteristics seen here are similar to the performance reported in the literature on state-of-the-art water electrolyzers.^43,44^ A more comprehensive comparison of obtained performance with state-of-the-art levels considering operational temperature, typical current densities, and some differences in MEA materials is given in Table S9.

**Figure 3:**
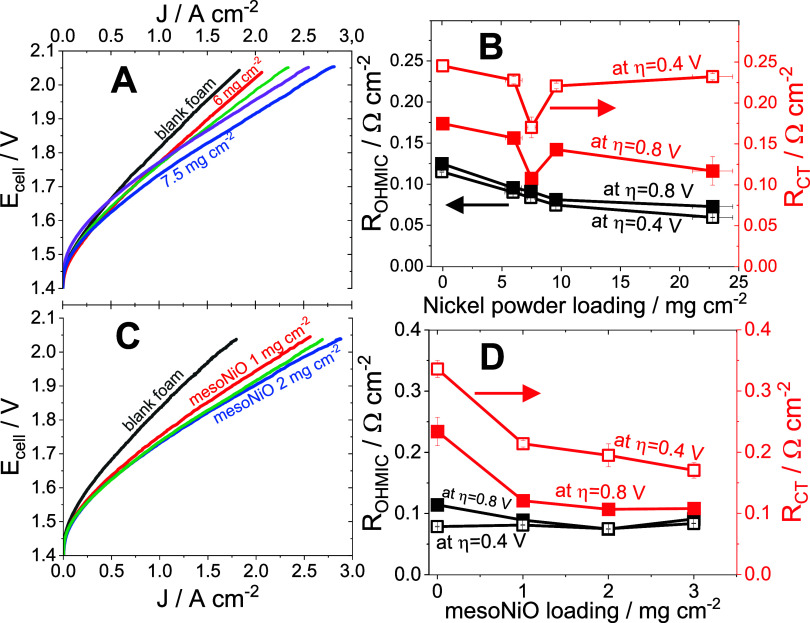
Operational characteristics of AEMWE. Close to steady-state voltammograms (1 mV s^–1^) obtained on AEMWE with different loadings of nickel powder ((A): constant mesoNiO loading 3 mg cm^–2^, nickel powder loading of 0.0–22.8 mg cm^–2^) and different loadings of mesoNiO ((C): constant nickel powder loading 7.5 mg cm^–2^, mesoNiO loading of 0–3 mg cm^–2^); the dependences of ohmic resistance and charge transfer resistance of AEMWE in operation on the loading of nickel powder ((B): constant mesoNiO loading 3 mg cm^–2^) and on the loading of mesoNiO ((D): constant nickel powder loading 7.5 mg cm^–2^) estimated from in situ EIS.

The thermodynamically dictated limit, the so-called Nernstian limit (1.229 V at 25 °C and 1.199 V at 60 °C), needs to be overcome to initiate the heterogeneous electron transfers in the course of the overall reaction (2H_2_O→O_2_ + 2H_2_) resulting in the appearance of faradaic currents. The kinetic region of the cell voltage is characterized by low currents (between 1 μA cm^–2^ and 1 mA cm^–2^) generated by the heterogeneous electron transfer, which is slower than the diffusion of reagents or products of the overall reaction. In contrast to the diffusion of products, the diffusion of reagents in both reactions 1 and 2 by the Grotthuss mechanism is faster than electron transfer. This implies that the currents recorded in the kinetic region originate only from the slow heterogeneous electron transfer. An increase in the driving force on AEMWE results in a change from the kinetic regime of slow heterogeneous electron transfer to the regime of linear proportionality between the current density and cell voltage, the so-called ohmic behavior, visible at current densities >0.5 A cm^–2^ (Figure 3A,C).

The difference between the Nernstian limit (*E*_Nernstian_) and the cell voltage (corrected on the ohmic drop) required for observation of the kinetic region, the so-called overpotential (η = (*E*_cell_ – *E*_Nernstian_)^*iR*−corrected^), is the voltage loss in the kinetic region of AEMWE operation that appears due to the slow heterogeneous electron transfer. The role of the catalyst is to reduce the voltage loss in the kinetic region.

The kinetic regime of AEMWE operation is manifested with an exponential increase in currents upon the increase in cell voltage, which is evident from the clear linearity in Tafel coordinates (Figure 4). Regardless of the nickel powder loading, the polarization curves obtained on AEMWE (Scheme 1G,H) and plotted in Tafel coordinates (Figure 4) showed the appearance of linearity, with a Tafel slope of ∼53 mV per decade of current density. An increased cell voltage resulted in the bending of the kinetic current of AEMWE, where the slope of 120 mV per decade of current can be distinguished.

**Figure 4:**
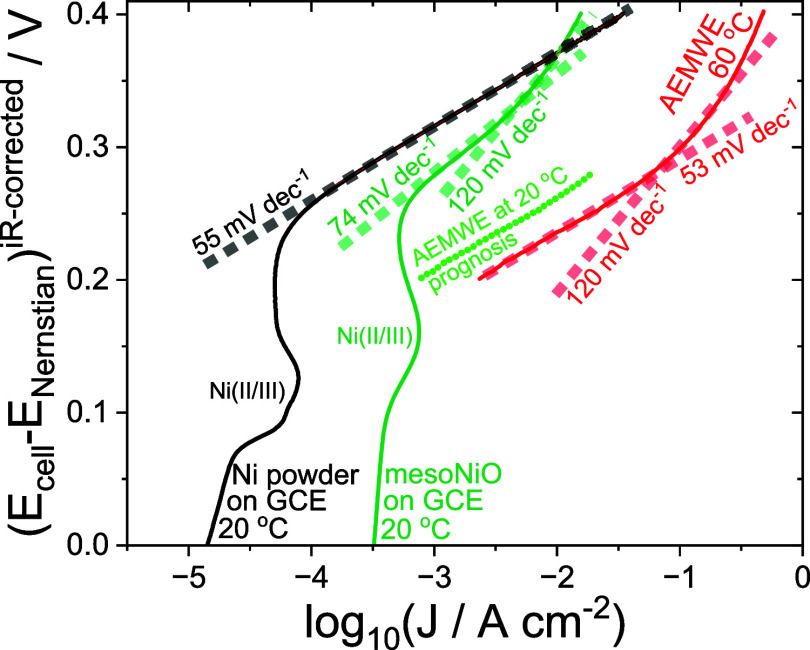
Tafel plots for close to steady-state linear voltammograms obtained on AEMWE (red curve: 60 °C, loading 3 mg of mesoNiO on 1 cm^–2^ of nickel foam) and on glassy carbon modified by nickel powder (0.1 mg cm^–2^) or by mesoNiO (0.4 mg cm^–2^). Dashed lines illustrate Tafel slopes. The green dotted line illustrates the temperature-corrected kinetic current on AEMWE.

To evaluate the individual contributions of the anode components, we performed CV measurements in a three-electrode cell filled with 1 M KOH on GCEs modified either by nickel powder or mesoNiO (Scheme 1A,B). The Tafel slope in the well-resolved kinetic region for the OER on nickel powder maintained over a few orders of magnitude of current was ∼55 mV dec^–1^ (Figure 4). The Tafel slope for the OER on mesoNiO was ∼77 mV dec^–1^, followed by curving toward higher values, up to 120 mV dec^–1^ at the high current densities. The steady-state measurements allowed the observation of the Tafel slope value for the OER on mesoNiO close to 60 mV dec^–1^.^45^

The Tafel slope value represents only the rate-determining step of the faradaic process (reactions 1 and 2). The assumption that the transfer coefficient (α) is equal to 0.5 ensures that the Tafel slope value is not random, as the steps of the electrode process adhere to a specific, well-defined mechanism.^46^ Under this important assumption, the computed values of the Tafel slope are strict^47^ (for OER: 21–22, 30, 40, 60, and 120 mV dec^–1^; for HER: 30, 40, and 120 mV dec^–1^). The condition of the strict values of the Tafel slope implies that all values observed in experiments on the three-electrode cell (Scheme 1A,B) and on AEMWE (Scheme 1G,H) reflect the unified value of 60 mV dec^–1^. Noticeably, the Tafel slope of 60 mV dec^–1^ is not attributed to any single-site mechanism of the HER,^47^ which supports the limitation of AEMWE by oxygen anode driving OER (reaction 2).

The Tafel slope of 60 mV dec^–1^ is assigned to the specific mechanism of the OER with the rate-determining step located on the chemical (electrochemistry-free) step, namely, deprotonation of the oxyhydroxide (NiOOH) intermediate^47^

3 $$\mathrm{NiOOH}+OH^{-}\to \mathrm{NiO}O^{-}+H_{2}O$$

The Tafel slope of 60 mV dec^–1^ observed in all systems of study illustrates that both the rate-determining step in reaction 3 and the mechanism of the OER are identical in three-electrode cells and in AEMWE.

Assuming the Arrhenius-type temperature dependence of the rate constant of heterogeneous electron transfer, it is possible to predict the kinetic current on AEMWE at room temperature for comparison with OER kinetic currents on the OER on nickel powder and mesoNiO (Figure 4). Indeed, OER kinetics on the anode of AEMWE is improved in comparison with the kinetics on individual catalysts, which could be articulated as ‘catalysis by the environment’. This is illustrated by both higher temperature-corrected kinetic currents and a 70 mV decrease in the overpotential achieved on AEMWE.

Modulation of the loading of both catalytic components, nickel powder and mesoNiO, had a minor effect on the kinetic region of the AEMWE anode process (Figures S8 and S9). Increased nickel powder loading had a negligible effect on the overpotential at 0.01 mA cm^–2^ (Figure S8B) and gave a minor decrease in the kinetic current density (at overpotential 0.24 V, Figure S8C). To consider the effect of the surface area expansion due to an increased nickel powder loading, we normalized the kinetic current density from AEMWE (Scheme 1H, Figure S8C) on the EASA-associated mass-normalized capacitive currents (from Figure 2B) obtained for electrodes in a three-electrode cell (Scheme 1D). An increased nickel powder loading led to a clear decrease in the EASA-normalized kinetic current density (Figure S8D), which confirms the absence of AEMWE performance improvement in the kinetic region. Similarly, the increased mesoNiO loading gave no improvement in AEMWE performance (Figure S9).

Increases in the loading of both nickel powder and mesoNiO on the anode resulted in increased current densities (Figure 5A,B) at a high cell voltage. Specifically, the best performance obtained on an AEMWE assembled with a compressed nickel foam anode loaded with 7.5 mg cm^–2^ nickel powder and 3 mg cm^–2^ mesoNiO was the following: current density 2.6 A cm^–2^ at cell voltage 2 V. The reproducibility of AEMWE characteristics is illustrated by the consistency of performance data reported by us previously for a similar system.^41^ Importantly, the best performance characteristics reported here are competitive with those reported for state-of-the-art PEMWE assembled on perfluorinated hydrocarbon membranes using noble anode catalysts (see Table S9).^48^

**Figure 5:**
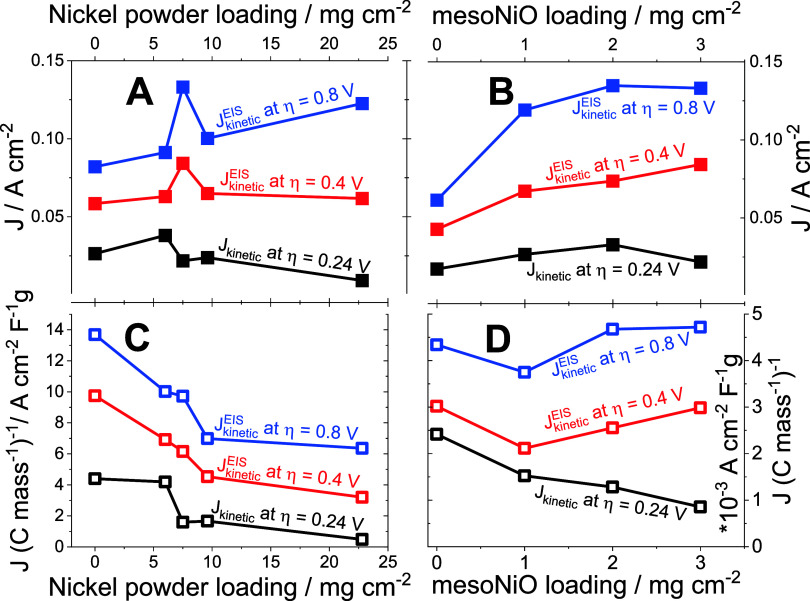
Dependence of the AEMWE anode kinetics on the catalyst loading. Dependences of kinetic currents on the loading of nickel powder ((A): mesoNiO (3 mg cm^–2^) is loaded on top of the nickel powder layer) and on the loading of mesoNiO ((B): nickel powder (7.5 mg cm^–2^) is loaded under the mesoNiO layer). (C, D): Dependences of the EASA-normalized kinetic currents on the catalyst loading.

Herein, we have now demonstrated that the performance of the device based on a membrane free from perfluorinated hydrocarbons and anode catalysts free from platinum group metals is competitive, thus implying an improvement in the sustainability of green hydrogen technologies.^44^

To evaluate the kinetics of the OER under operational conditions, we utilized in situ potentiostatic EIS at the cell voltages 1.6 and 2.0 V (overpotentials 0.4 and 0.8 V, respectively). Such conditions correspond to the practical current density range for the industrial application of AEMWE (1.0–2.5 A cm^–2^).^5^ The fitting of experimental spectra (Supporting Note 3) was carried out using an equivalent circuit, which enables the estimation of the charge transfer resistance, illustrating the heterogeneous electron transfer (*R*_CT_). The experimental and theoretical spectra presented in Nyquist coordinates fit well (Figure S11). The high-frequency inductive loop observed for the experimental EIS spectra (Figure S11) is assigned to the stray impedance of the testing hardware.

The effect of smoothing of compressed nickel foam by nickel powder loading on the ohmic resistance of AEMWE in operation is visible as a decrease in the ohmic resistance (Figure 3B,3D), which is almost independent of the operational voltage. A slight increase of *R*_ohm_ at the overvoltage of 0.8 V is attributed to the intense bubble evolution and somewhat restricted electrolyte access to the membrane.^49^ The values of the ohmic resistance are the sum of the interfacial contact resistances between different components of the cell as well as the bulk resistances inside the components.^50^ The nickel powder loading of 22.8 ± 1.7 mg cm^–2^ enabled us to achieve a *R*_ohm_ value of ∼60 mΩ cm^2^, which presents the minimized ohmic loss on AEMWE operation; furthermore, it is lower than published values (up to 300 mΩ cm^2^).^35^

In contrast to the ohmic resistance, the charge transfer resistance exhibited a clear decrease upon an increase in cell voltage (Figure 3B,D). This motivates its assignment to heterogeneous electron transfer during the OER. Increases in the loading of both catalysts (nickel powder and mesoNiO) lead to a decrease in charge transfer resistance.

Assuming the absence of the diffusion limitation on AEMWE, at the high overpotentials, the apparent kinetic current density can be estimated by eq 4

4 $$J_{kinetic}^{EIS}=\frac{\mathrm{RT}}{\alpha \mathrm{nF}R_{CT}}$$

where α is the transfer coefficient (assumed to be 0.5), *R* is the gas constant (8.314 J mol^–1^ K^–1^), *T* is the absolute temperature (333 K), *n* is the number of electrons (four for the overall process 2H_2_O→O_2_ + 2H_2_), and *F* is the Faraday constant (96485 C mol^–1^). Consistently with current densities observed at the purely kinetic region (low overpotential 0.24 V), the kinetic current densities (*J*_kinetic_^EIS^) estimated at higher overpotentials (0.4 and 0.8 V) showed a clear increase with an increase in the overpotential (Figure 5A,B), which supports their assignment to the heterogeneous electron transfer.

Importantly, the increase in kinetic current density upon increased nickel powder loading (with the mesoNiO loading held constant) is evident for AEMWE operation at high cell voltage (Figure 5A), implying improved OER kinetics. Operation at the lower overpotential (0.4 V) and in the purely kinetic regime showed the absence of the effect of nickel powder loading. Similar behavior is evident for an increased mesoNiO loading (with the nickel powder loading held constant) (Figure 5B).

In contrast to mesoNiO loading behavior on AEMWE, increasing the mesoNiO loading on smooth glassy carbon, evaluated in a three-electrode cell (Figure S13A, Scheme 1B), revealed nonmonotonic dependencies for both EASA (Figure S13B) and faradaic OER currents at an overpotential of 0.4 V (Figure S13C). Still, a clear correlation between OER kinetic currents and EASA was observed (Figure S13D). This suggests that at high loadings, a portion of mesoNiO on a smooth electrode becomes inaccessible for both the EASA process and the OER due to its high surface area. A similar limitation in catalyst surface accessibility for the OER at smooth glassy carbon was observed in microporous NiO,^39^ which has up to 5 times higher surface area than mesoNiO. Importantly, increasing the mesoNiO loading on the AEMWE anode led to a noticeable rise in apparent kinetic current at the same overpotential (0.4 V, Figure 5B). This suggests that even with a 10-fold increase in loading, mesoNiO remains actively involved in the electrode process, indicating efficient electron transfer from the catalyst to nickel. The improved utilization of mesoNiO on the AEMWE anode could be attributed to both the high mechanical compression applied on the catalyst during AEMWE assembly and the roughened surface of nickel foam modified with nickel powder.

To determine the effect of the EASA on the kinetics of the AEMWE anode process, we normalized the current densities estimated at the different overpotentials on AEMWE (Figure 5A,5B) on the experimental descriptor of EASA, namely, mass-normalized capacitances of the electrical double layer (from Figure 2B,2D, respectively). The clear decrease of all EASA-normalized currents is evident for increased loadings of both nickel powder (Figure 5C) and mesoNiO (Figure 5D). This implies that the modification of the anode by both catalysts improves the AEMWE performance only by increasing the surface area hosting a faradaic process. However, the modification does not improve the catalytic capability of the anode.

### 3.3 Surface Analysis of Catalysts

The state of the catalytic surfaces and the changes imposed by the OER were characterized by surface analysis; XPS and XAS analysis of pristine and post-mortem AEMWE anodes were carried out. The organic component present within the catalyst layers (Nafion ionomer) interferes with the analysis of oxygen and carbon. We therefore focused only on the elemental analysis of transition metals.

The possible contamination of the anode with iron species, which are inherently present in electrolyte fed and could appear due to the leaching from the stainless-steel test cell flow fields, leads to a significant overestimation of anode performance.^51^ However, the evaluation of the post-mortem anode on the presence of iron species by XPS showed the absence of visible iron peaks at 711 and 724.5 eV (Figure S14). This excludes the possible effects of iron contamination from the observed phenomena on AEMWE.

Core-level Ni 2p high-resolution XPS spectra (Figure 6A,B) were acquired for pristine samples of compressed nickel foam, nickel powder, and mesoNiO, as well as for AEMWE anodes (Scheme 1H, compressed nickel foam loaded with nickel powder (7.5 mg cm^–2^) and mesoNiO (3 mg cm^–2^)) before (pristine) and after (post-mortem) device operation.

**Figure 6:**
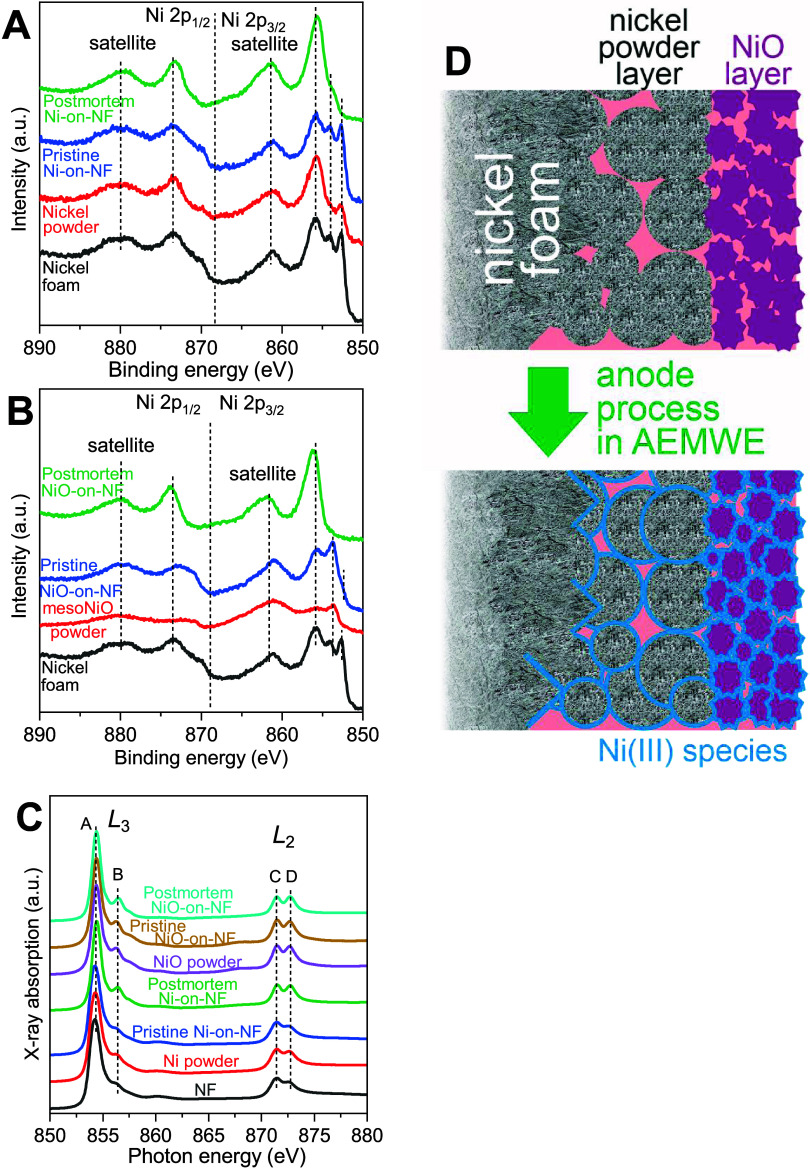
Oxidation of all components of the AEMWE anode by the OER. High-resolution XPS spectra of the Ni 2p core level acquired on pristine and post-mortem samples (A, B); (C): Ni L-edge XAS spectra. (D): Schematic of oxidation of all nickel-based components of the AEMWE anode.

Here, we take Biesinger’s report on the analysis of mixed nickel metal, oxide, and hydroxide as a reference, which matches our system well.^52^ All of our spectra showed a good Ni 2p feature, having two regions of Ni 2p_1/2_ and Ni 2p_3/2_ due to spin–orbit splitting and the main peaks accompanied by satellite peaks. It is clear that pristine nickel foam has features of metallic nickel and nickel­(II) in the form of oxide, assigned to the lowest sharp peak at ∼852 eV and double peaks located at 855 eV, respectively (Figure 6A).^52,53^ This demonstrates a coexistence of Ni(0) and Ni­(II) species because of partial surface oxidation.

Differently, XPS spectra recorded for nickel powder show one main peak located at ∼857 eV in addition to the one assigned to Ni(0), which implies the presence of a higher oxidation state of nickel, Ni­(III). The higher oxidation state visible on the powder but not on the foam may be due to the high surface area at the nanoscale compared with the bulk nickel foam. This could be responsible for the noticeable OER activity of nickel powder compared to NiO (e.g., Figure 4). A comparison of XPS spectra of pristine and post-mortem anodes showed a significant change in the state of nickel, from multiple peaks to one main peak, which indicates the transformation from Ni(0) and Ni­(II) to Ni­(III) (Figure 6A). This suggests a more severe oxidation after the OER to form oxyhydroxide NiOOH on the surface.

On the other hand, XPS spectra of mesoNiO powder and a pristine anode based on mesoNiO-loaded nickel foam showed NiO features. A similar spectrum was observed for a post-mortem mesoNiO-based anode. Both nickel foam and NiO were oxidized to the Ni­(III) species on the surface, exhibiting only one peak at ∼857 eV (Figure 4B).

To confirm the chemical state of the surface and look deeper into the material, we also conducted XAS measurements. The shapes of the recorded spectra (Figure 6C) were consistent for selected samples, with two regions, the *L*_3_ edge located at 855 eV and the *L*_2_ edge centered around 872 eV. Two regions have two peaks due to the crystal field effect of the local environment.^54^ The fingerprint of double peaks (A and B) in the *L*_3_ region suggests the oxidized state of Ni atoms in all samples, indicating the surface oxidation of nickel powder and compressed nickel foam. Peak B showed higher intensities on mesoNiO and on pristine mesoNiO-on-nickel foam (Table S8) compared to the nickel foam, nickel powder, and pristine nickel-on-nickel foam, indicating higher oxidation. A clear increase of peak B intensity was observed on post-mortem nickel-on-nickel foam in comparison with pristine one, indicating a transformation from Ni­(II) to Ni­(III) after the OER.^55,56^ Such an increase is not visible for pristine and post-mortem NiO-on-NF anodes. This could be due to the inhomogeneous catalyst layer, resulting in a lowering of the Ni­(III) signal compared to the pristine samples. The consistency of XPS and XAS data implies that the OER leads to severe oxidation on all nickel components of the anode: nickel foam, nickel powder, and mesoNiO. The oxidation process leads to the formation of NiOOH on the surface as an OER intermediate (Figure 6D). This agrees with the analysis of the OER mechanism by Tafel slope estimation (Figure 4). The formation of oxyhydroxide NiOOH as a reaction intermediate has been widely reported for OER catalysts based on transition metals.^55^

## 4 Conclusions

Herein, we investigated the effects of the loading of nickel powder and mesoNiO on the kinetics of the anode process in alkaline media at the conditions of half-cell (three-electrode cell) and operational AEMWE. In the kinetic region, a current density up to 0.1 A cm^–2^ on an AEMWE revealed the participation of both nickel and mesoNiO in the OER catalysis, and there was also an improvement of the heterogeneous electron transfer rate, in comparison with the rate of identical processes on individual catalysts. Optimization of the loading for both mesoNiO and nickel powder enabled us to achieve a current density of 2.6 A cm^–2^ at cell voltage 2 V. This is comparable with the state-of-the-art performance achieved on water electrolyzers based on perfluorinated membranes and noble catalysts. The improvement of the AEMWE performance is due to the increased EASA of the anode. Post-mortem surface analysis of the anodes confirmed the involvement of all electrode components in the catalysis of the anode process.

The engagement of all nickel components in the catalysis of the OER opens a route toward catalyst-free anodes in alkaline water electrolyzers as a strategy to improve the sustainability of green hydrogen technologies by the avoidance of binders based on perfluorinated ionomers (e.g., Nafion). Furthermore, the cost of the materials will be reduced.

## Supplementary Material



## Notes

AEMWE test bench schematic; additional SEM images; additional experimental details on the estimation of total capacitance by EIS; mesoNiO characterization, parameters of EIS fitting; additional data on the loadings of nickel powder and mesoNiO on AEMWE performance; description of in situ EIS on AEMWE; Nyquist plots; description of equivalent circuits applied to AEMWE data fitting, additional XPS and XAS data, and the comparison of obtained performance with the relevant data for AEMWE and PEMWE (PDF)

## ○○

SUNCAT Center for Interface Science and Catalysis, National Accelerator Laboratory, Mento Park, CA 94025, USA. Department of Chemical Engineering, Stanford University, Stanford, CA 94305, USA
