# Supplementary material for: Advanced Nickel-Based Gas Diffusion Anode for Zero-Gap Anion-Exchange Membrane Water Electrolyzers
Source: ACS Appl Mater Interfaces. 2025 May 23;17(22):32216–27. doi: 10.1021/acsami.5c01272 (PMC12147071; doi:10.1021/acsami.5c01272)
Supplement: Supplementary file 1 [file am5c01272_si_001.pdf]

SUPPORTING INFORMATION

Advanced nickel-based gas diffusion anode for zero-gap anion-exchange membrane water electrolyser

Irina V. Pushkareva[#a*], Zhixing Wu[#,b], Xianjie Liu[b], Maksim A. Solovyev[c,d], Sergey I. Butrim[c,d], Margarita V. Kozlova[c,d], Tatiana L. Kulova[d,e], Reverant Crispin[b,g,h], Emma M. Bjork[f], Dmitri G. Bessarabov[a], Mikhail Vagin[b,g*], Artem S. Pushkarev[a]

[a]HySA Infrastructure Center of Competence, Faculty of Engineering, North-West University, Private Bag X6001, Potchefstroom Campus 2531, South Africa

[b]Laboratory of Organic Electronics, Department of Science and Technology (ITN), Linköping University, 60174 Norrköping, Sweden

[c]National Research Center "Kurchatov Institute" 1, Kurchatov sq., Moscow, 123182, Russia

[d]National Research University "Moscow Power Engineering Institute", 14, Krasnokazarmennaya str., 111250 Moscow, Russia

[e]Frumkin Institute of Physical Chemistry and Electrochemistry, Russian Academy of Sciences, 31-4 Leninskii ave., 119071 Moscow, Russia

[f]Nanostructured Materials, Department of Physics, Chemistry and Biology (IFM), Linköping University, SE 58183 Linköping, Sweden

[g]Wallenberg Initiative Materials Science for Sustainability, Department of Science and Technology, Linköping University, 60174 Norrköping, Sweden

[h]Wallenberg Wood Science Center, Linköping University, 60174 Norrköping, Sweden

#Authors have contributed equally

*Corresponding authors:

Irina V. Pushkareva, pushkareva_iv@outlook.com, 50161067@mynwu.ac.za

Mikhail Vagin, mikhail.vagin@liu.se, +46702753087

Table of Contents

Figures / Tables / Notes	Page
1. **Figure S1**	3
2. **Figure S2**	4
3. **Supporting Note 1**	5
4. **Figure S3**	6
5. **Figure S4**	7
6. **Supporting Note 2**	8
a. **Figure S5**	8
b. **Table S1**	8
c. **Figure S6**	9
7. **Supporting Note 3**	10
a. **Figure S7**	11
b. **Table S2**	12
8. **Figure S8**	13
9. **Figure S9**	14
10. **Supporting Note 4**	15
a. **Figure S10**	16
b. **Figure S11**	20
c. **Figure S12**	21
d. **Table S3**	22
e. **Table S4**	22
f. **Table S5**	23
g. **Table S6**	24
h. **Table S7**	25
11. **Figure S13**	26
12. **Figure S14**	27
13. **Table S8**	28
14. **Table S9**	29

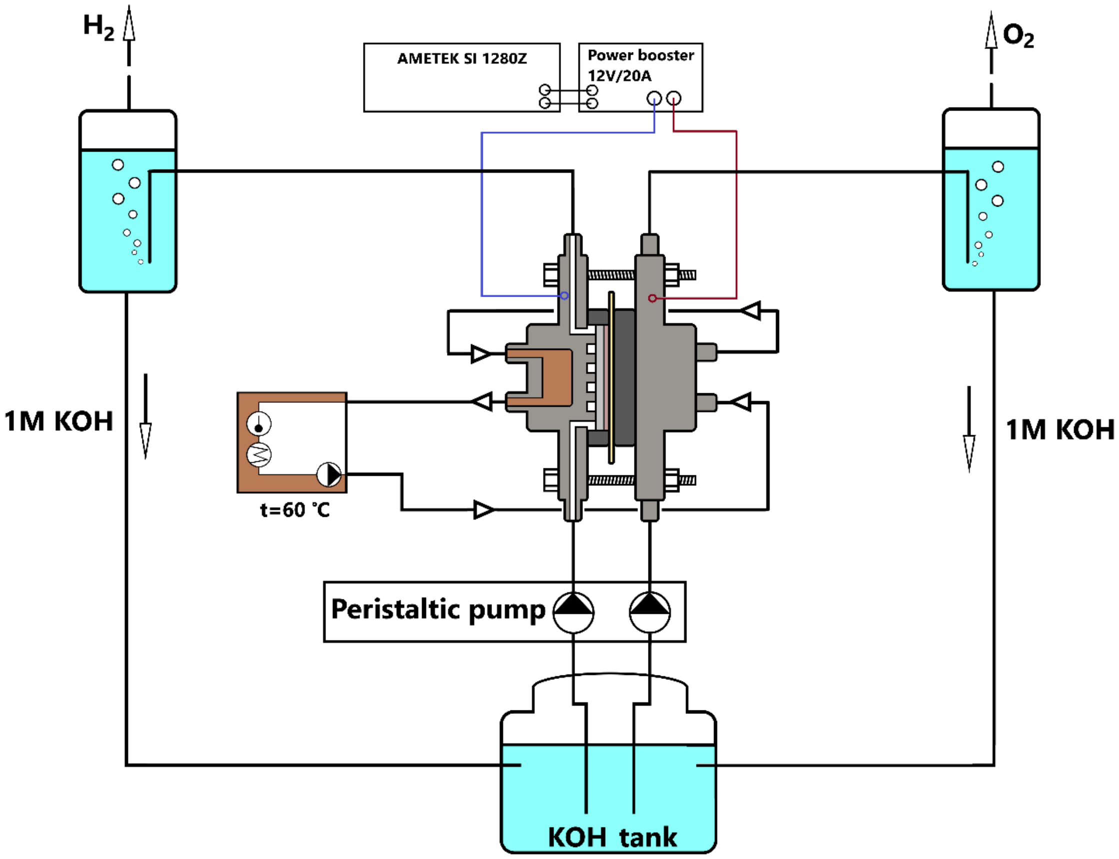

Figure S1. Schematic of the test bench for an AEMWE.

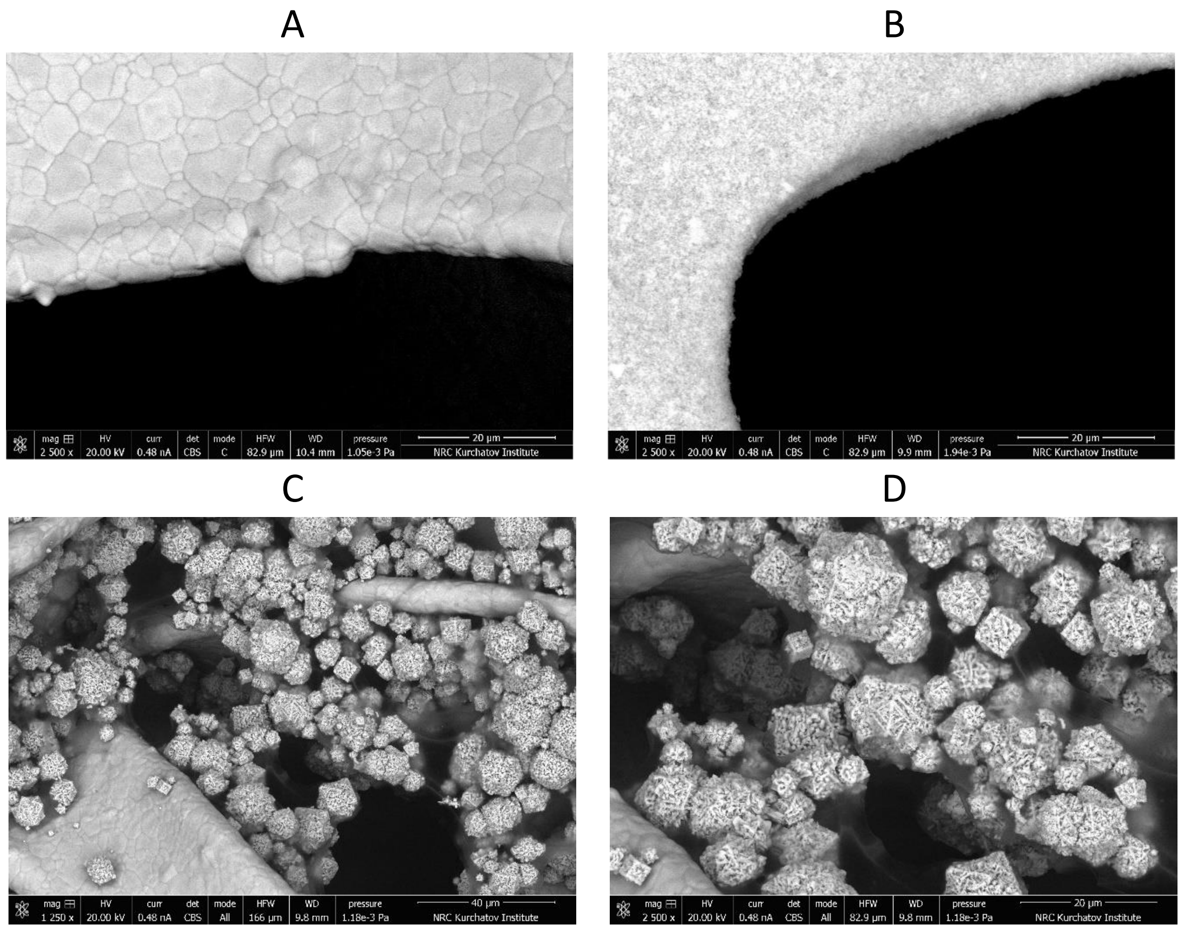

Figure S2. SEM images of nickel foam: bare (A), modified by mesoNiO (B), and modified by nickel powder at lower (C) and higher (D) magnification.

Supporting Note 1.

Estimation of the total capacitance by EIS

The total capacitance was calculated for each individual frequency using imaginary parts of impedance normalised by angular frequency, as follows:

$$C_{TOTAL} = \frac{Y''}{\omega} = \frac{Z''}{2\pi\upsilon((Z')^2 + (Z'')^2)}$$

where Z'' and Y'' are the imaginary parts of impedance (Ω) and admittance (Ω^{-1}), respectively, and υ is the frequency (Hz). The plots of total capacitance vs frequency (Fig. S3A) show the characteristic transition from the near-vertical frequency-independent region to the frequency-dependent part of the spectra. The region of such transitions could be used as an estimate of the total capacitance.

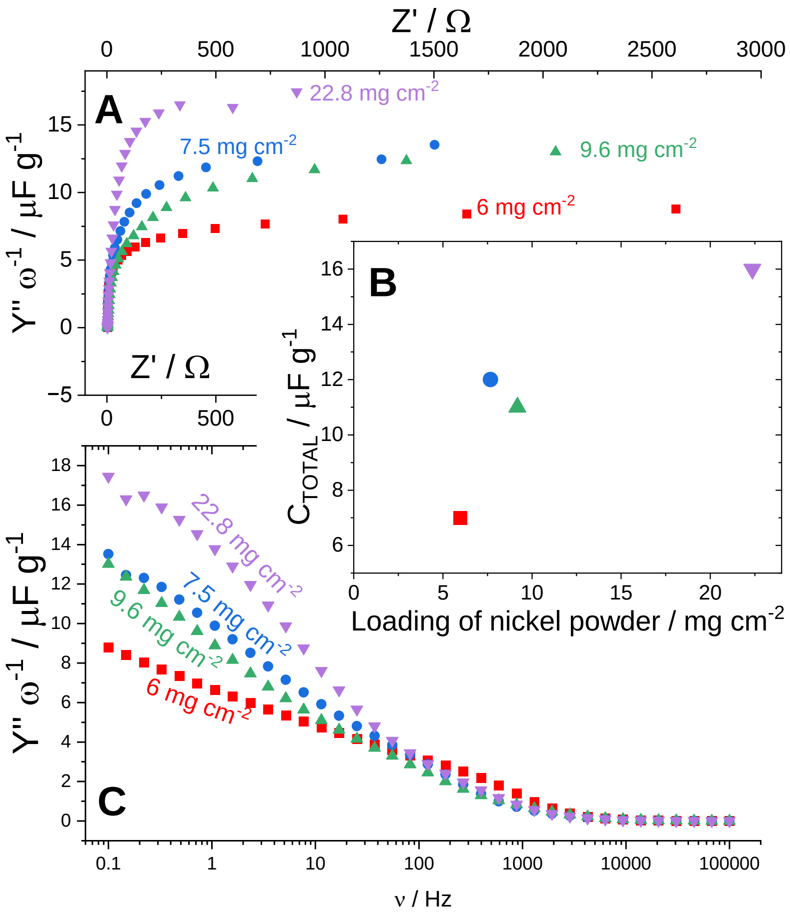

Figure S3. Increase in EASA of nickel foam by modification with nickel powder. **A:** impedance spectra of the electrodes (Scheme 1C–E) in complex capacitance coordinates; **B:** dependence of the total capacitance estimated from Fig. S3A on the nickel powder loading; **C:** frequency dependence of the frequency-normalised admittance.

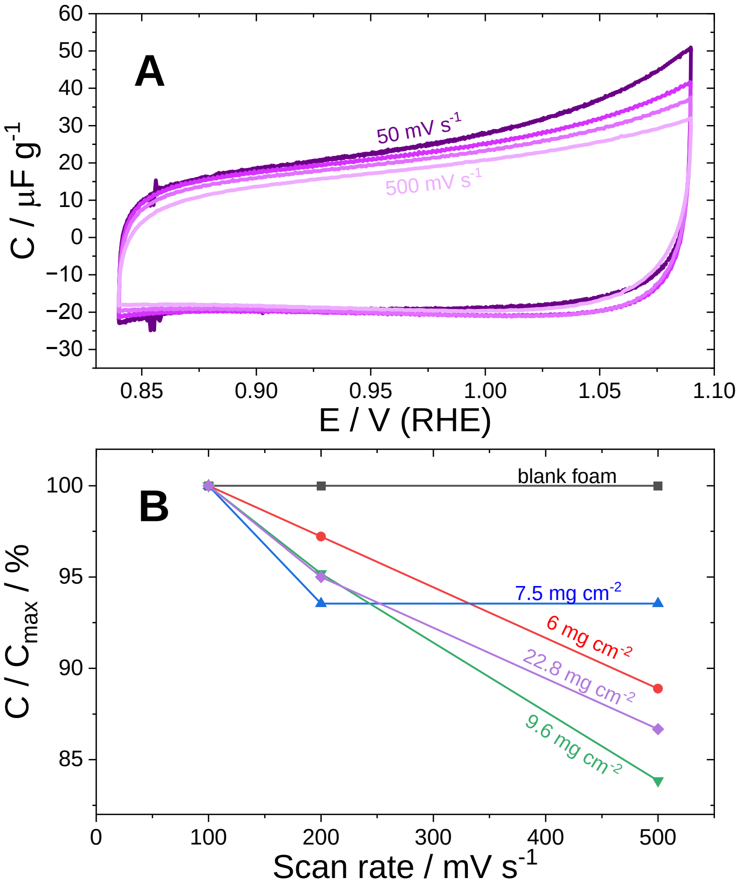

Figure S4. Ionic transport limitation on nickel foam modified by nickel powder. **A:** scan rate-normalised cyclic voltammograms obtained on nickel foam electrode modified by nickel powder (Scheme 1D, loading 22.8 mg cm^{-2}); **B:** scan rate dependence of the maximum normalised capacitance.

Supporting Note 2.

Characterisation of mesoNiO

XRD patterns of synthesised mesoNiO (Fig. S5(a)) have diffraction peaks that are well assigned to cubic NiO (PDF cards No. #047-1049). BET analysis of mesoNiO shows type II isotherms with a hydrolysis loop, offering evidence of the presence of mesoporosity [1] (estimated pore size ~3 nm (Fig. S5(b) and S5(c)); specific surface area 126.8 m^2 g^{-1} and pore volume 0.27 cm^3 g^{-1}). The mass-normalised double layer capacitance of mesoNiO is 10 times greater than that of metallic nickel (1.6 F g^{-1} and 15 F g^{-1} for nickel powder and mesoNiO, respectively: Fig. S6).

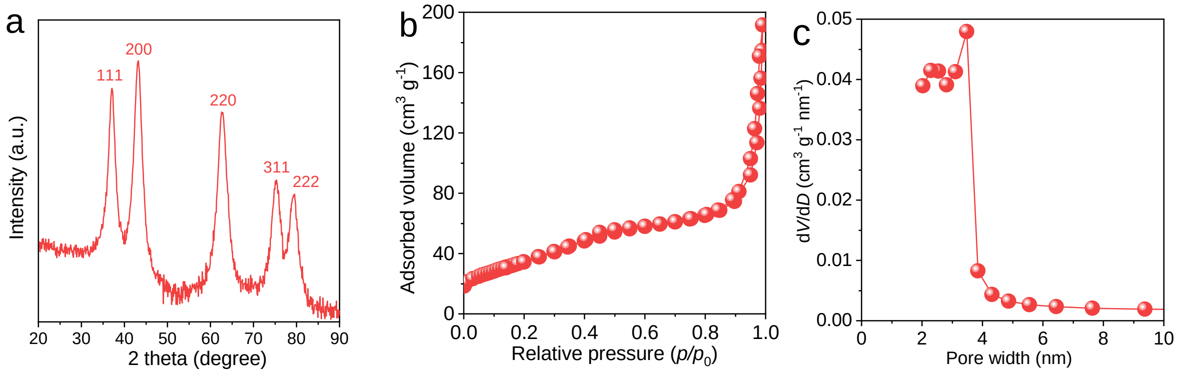

Figure S5. Synthesised mesoNiO: (**a**) XRD pattern, (**b**) physisorption isotherm and (**c**) pore size distribution.

Table S1. Synthesised mesoNiO: BET specific surface area, pore volume and pore size.

Sample	BET specific surface area / m^2 g^{-1}	Pore volume / cm^3 g^{-1}	Pore size / nm
mesoNiO	126.8	0.27	3.5

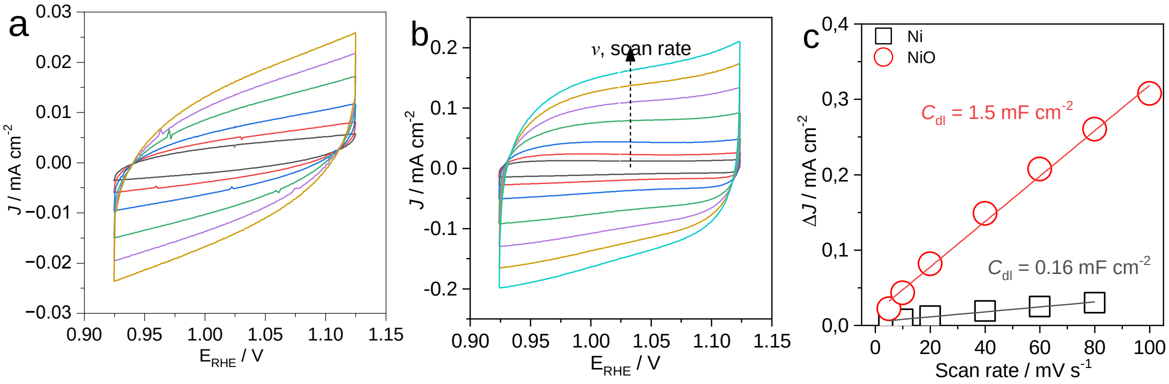

Figure S6. CV at different scan rates on GCE modified by (**a**) nickel powder (0.1 mg cm^{-2}) or by (**b**) mesoNiO (0.1 mg cm^{-2}). (**c**) Dependence of the capacitive current density (from **a** and **b**) on the scan rate.

Supporting Note 3.

Estimation of the total capacitance of mesoNiO deposited on nickel foam modified by nickel powder

Samples of modified nickel foams were used as working electrodes in a three-electrode cell filled with 1 M KOH (Scheme 1D-E) to collect the impedance spectra. The presence of two time-resolved processes visible in the Bode plot (Fig. S7B) was modelled by two Voight (parallel connection of *R* and *C*, *R*-*C*) elements in the simplest unified equivalent circuit (inset in Fig. S7A) developed for an electrode covered with a damaged (porous) coating [1,2]. Since the boundary between the layers is not ideally smooth, due to increased surface roughness of the porous film, a quantitative analysis of the electrode impedance response requires a more complicated, distributed circuit model featuring constant phase elements (CPE) rather than pure capacitors. The equivalent circuit (inset in Fig. S7A) providing the best fit consists of the solution resistance R_s and two combined *R-CPE* units (I and II). The capacitance raised through the whole film is represented by the film capacitance C_{film}, which, as we believe, represents the EASA capacitance. A single set of parameters was used to simultaneously fit the real and imaginary parts of the impedance over the frequency range 1.25 Hz to 50 kHz. A value of the fitting quality parameter χ^2 of ≤ 0.001 obtained for all spectra indicates a very good fit (Fig. S7A,B). The R_I-$C_I$unit showed smaller values than the second one, illustrating the faster kinetics of the first process (Table S2). Therefore, the R_{II}-C_{II} unit might be assigned to fast electronic transport contributed by faradaic phenomena (where R_I is the charge transfer resistance proportional to the revers rate constant of the electrode reaction and C_I is the double layer capacitance at the bottom of the large pores created on nickel foam), while the R_{II}-C_{II} unit might be assigned to the slower ionic transport (where R_{II} R_{II} is the resistance of the film established via pores and C_{II} is the double layer capacitance inside the high surface area mesoNiO) [1].

References:

[1] R.D. Armstrong, A.T.A. Jenkins, B.W. Johnson, An investigation into the UV breakdown of thermoset polyester coatings using impedance spectroscopy, Corros. Sci. 37(10) (1995) 1615–1625.

[2] M. O'Donoghue, R. Garrett, V. Datta, P. Roberts, T. Aben, Electrochemical impedance spectroscopy: Testing coatings for rapid immersion service, Mater. Perform 42(9) (2003) 36-41.

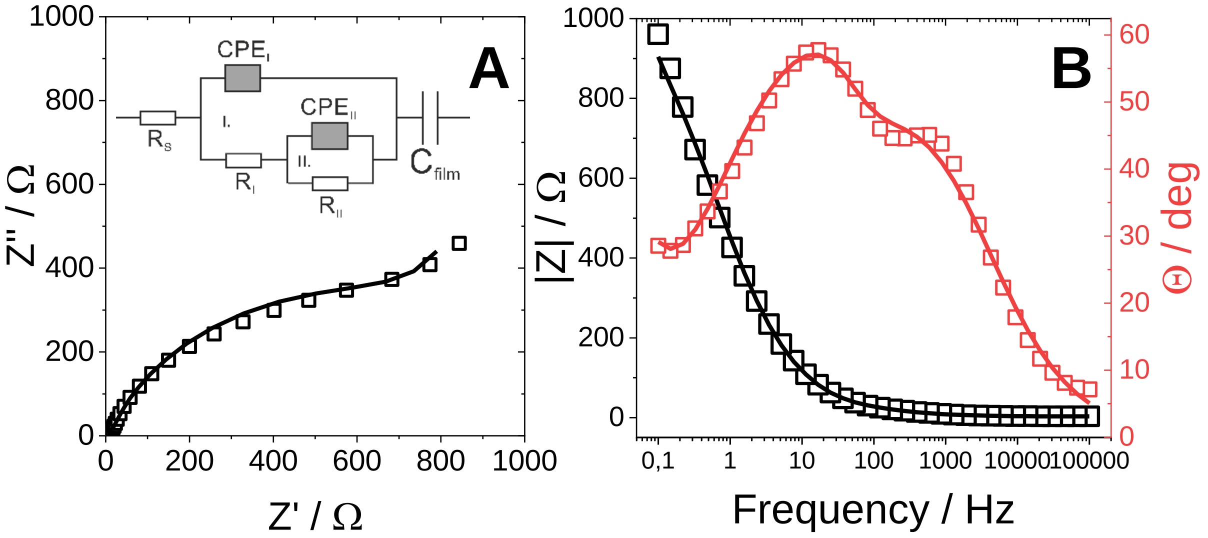

Figure S7. Example of impedance spectrum in Nyquist (**A**) and Bode (**B**) coordinates obtained on electrode (compressed nickel foam modifed firstly by nickel powder (7.5 mg cm^{-2}) and secondly by mesoNiO (3 mg cm^{-2})) in a three-electrode cell (Scheme 1E; 0.94 V (RHE), 10 mV amplitude, 1M KOH). Inset: the equivalent circuit used for fitting (open symbols and solid curves are experimental data and fitted spectra, respectivly).

Table S2. Parameters of impedance spectra fitting for nickel-modified electrodes (Scheme 1D,E).

	Nickel powder loading / mg cm^{-2}	MesoNiO loading / mg cm^{-2}	R_S Ω	P_I[1] ×10^{-4}	ϕ_I[2]	C_I[3] μF	R_I Ω	$R_I C_I$ ×10^{-6} s	P_{II} ×10^{-4}	ϕ_{II}[2]	R_{II} Ω	$R_{II}C_{II}$ ×10^{-2} s	C_{film} mF
1	7.67	0	1.98	3.30	0.737	24.2	1.635	39.6	0.28	1[4]	1364	3.79	3.19
2	7.67	1	3.16	1.03	0.811	15.8	107.4	1690	3.24	0.71	909.3	1.86[5]	4.44
3	7.67	2	2.94	4.91	0.653	15.2	149.1	2270	0.084	1[4]	876.1	4.46	7.25
4	0	3	2.91	6.96	0.640	21.2	16.22	343	0.19	1[4]	379.1	0.735	8.52
5	6	3	2.65	3.14	0.750	29.4	5.178	152	0.204	1[4]	1564	3.19	4.24
6	9.19	3	1.61	3.56	0.516	32.8	2.613	0.89	0.13	1[4]	823.5	1.11	6.45
7	22.36	3	2.45	4.30	0.707	25.1	3.409	85	0.23	1[4]	1433	3.25	3.47

[1]Fitted parameter of the CPE

[2]Fitted exponent factor, which varies from 0 to 1. When ϕ is tending to 0 the CPE behaves as a pure resistor and when ϕ is tending to 1 the CPE represents a pure capacitor

[3]Capacitance estimated as $C = \left(P \times (R_S)^{(1-\phi)}\right)^{1/\phi}$

[4]Pure capacitor, which implies $P_{II} = C_{II}$

[5]Where capacitance C_{II} is estimated as $C_{II} = \left(P_{II} \times (R_S)^{(1-\phi_{II})}\right)^{1/\phi_{II}}$

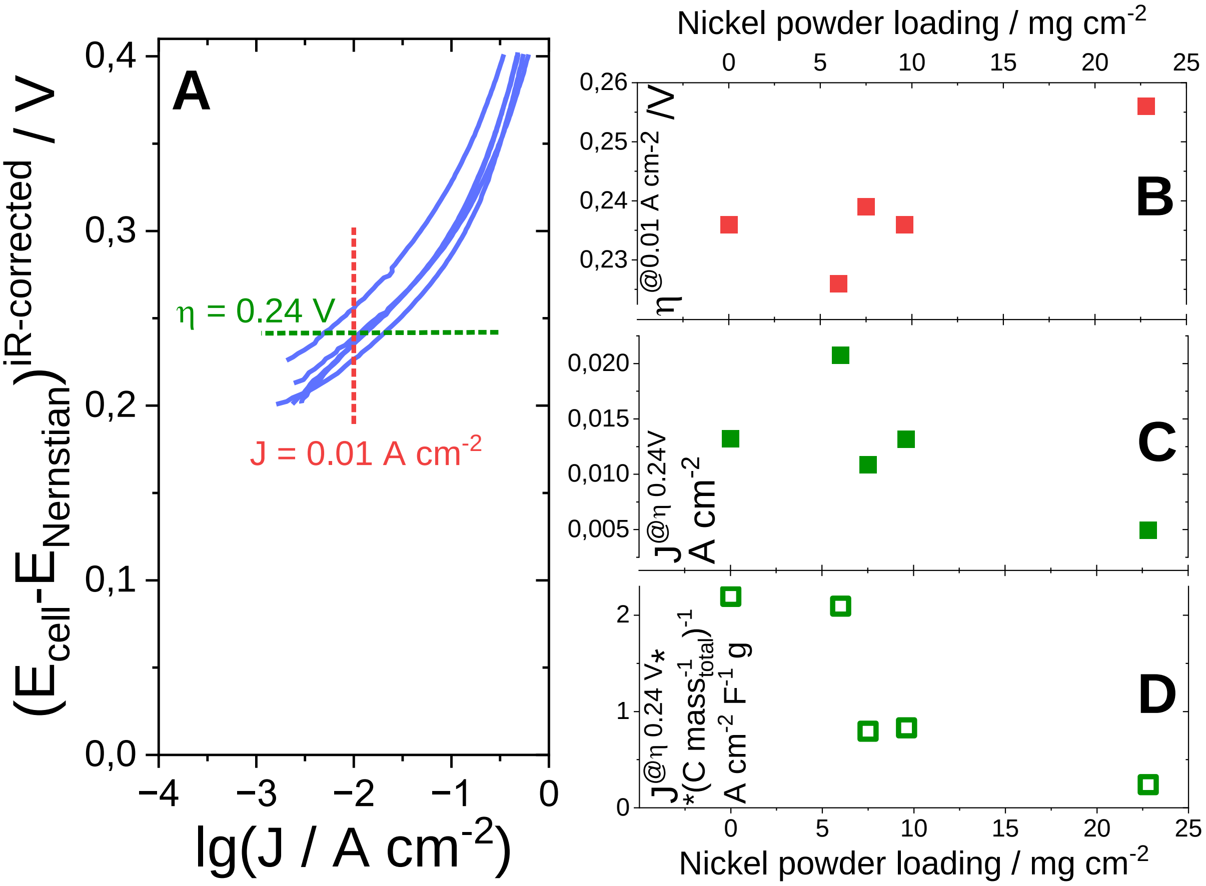

Figure S8. Absence of the loading effect of nickel powder on AEMWE performance in the kinetic region. **A:** Tafel plots of close-to-steady state polarisation of curves obtained on AEMWE with anodes of different nickel powder loadings (mesoNiO loading 3 mg cm^{-2}); the dependencies of overpotential (at 10 mA cm^{-2}, **B**), current density at overpotential of 0.24 V (**C**) and the EASA-normalised current density (**D**) on the nickel powder loading at AEMWE anode.

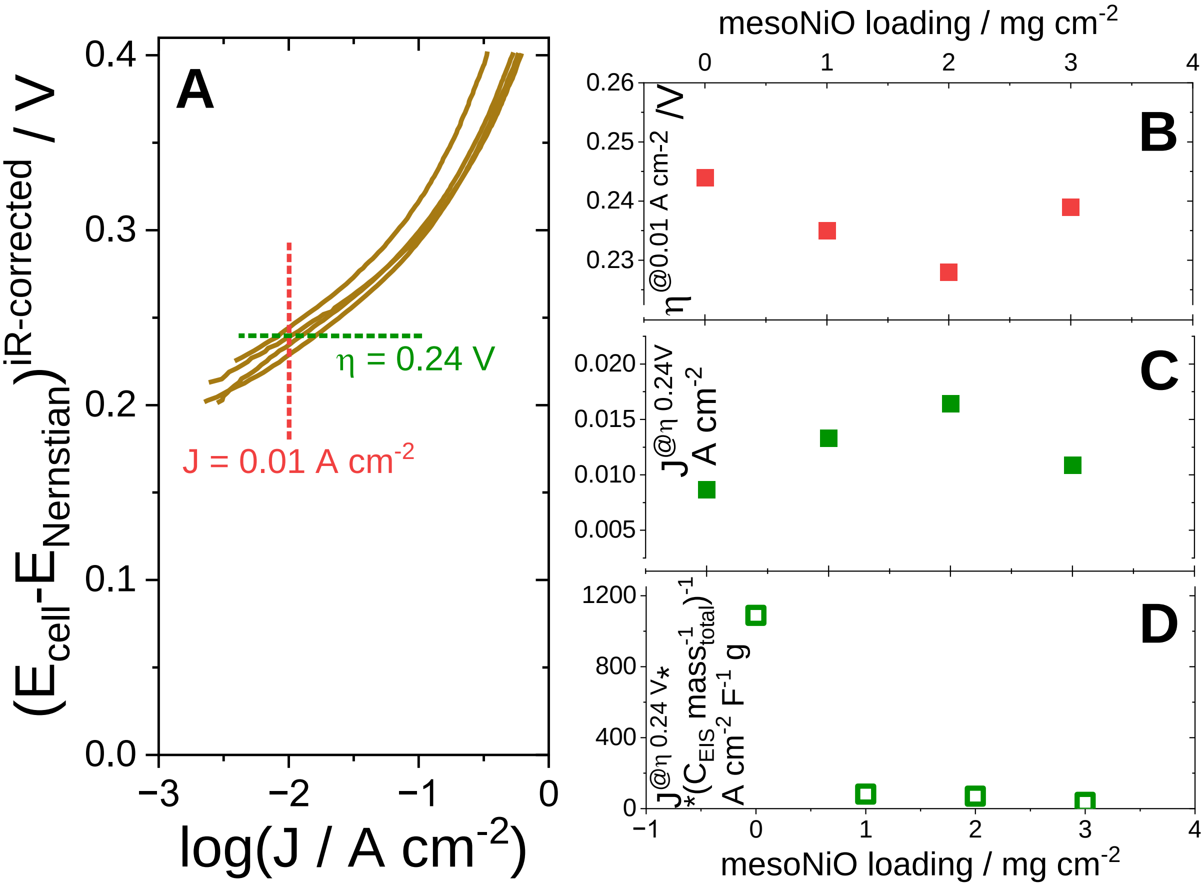

Figure S9. Absence of the mesoNiO loading effect on AEMWE performance in the kinetic region. **A:** Tafel plots of close-to-steady state polarisation of curves obtained on AEMWE with anodes of different mesoNiO loading (nickel powder loading 7.5 mg cm^{-2}); the dependencies of overpotential (at 0.01 A cm^{-2}, **B**), current density (at overpotential 0.24 V) (**C**) and the EASA-normalised current density (**D**, EASA is estimated from EIS measurements) on the mesoNiO loading at AEMWE anode.

Supporting Note 4

In situ EIS on AEMWE

The quality of the measured EIS data of the AEMWE should be evaluated in terms of its compliance to the linearity, causality and time invariance criteria (so called Kramers–Kronig, KK, criteria). The Lin-KK [2] software was used. The calculated residuals of the raw spectra are shown in Fig. S10. These are consistently very low and thus indicate valid impedance spectra in compliance with the linearity and time invariance criteria. Important to note is that residuals are generally < 2%. Higher residuals at higher frequencies emanate from stray impedance, which is rather difficult to be processed by the Lin-KK software due to its algorithm giving higher values of residuals.

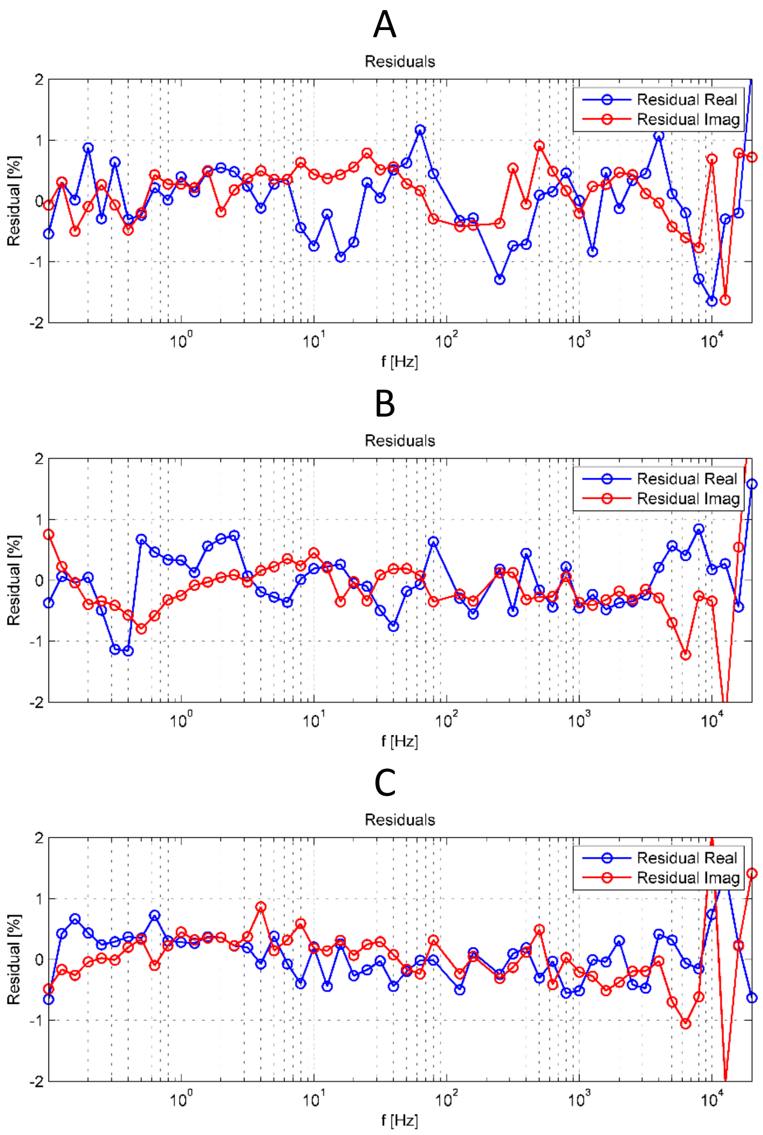

Figure S10. Example of calculated residuals of the spectra to evaluate the KK criteria complince, obtained using the Lin-KK software. The raw EIS data were collected on AEMWE at 1.45 (**A**), 1.6 (**B**) and 2.0 V (**C**) (60 °C, 1 M KOH, flow rate 3 ml min^{-1}). Nickel powder loading 7.5 mg cm^{-2}, mesoNiO loading 3.0 mg cm^{-2}.

Experimental EIS spectra (Fig. S11) feature an inductive behavior at both high and low frequencies. Recently, the pseudo-inductive feature has been reported for PEMWE [3] and AEMWE [4, 5], leading to an apparent decrease of the cell voltage and performance improvement. We addressed this peculiarity by introducing inductive elements into the general equivalent circuit utilised for fitting (Fig. S12A). The serial addition of the pseudo-inductive low frequency feature and the high-frequency stray impedance sub-circuit [6, 7] appeared from the potentiostat booster and/or the cell connection. The circuit is also based on a typical ohmic resistance R_{OHM} in series with three *R-CPE* elements to fit the time constant dispersion at medium to low frequencies, related to the charge transfer resistance [8]. Since the boundary between the layers is not ideally smooth, due to increased surface roughness of the porous film, a quantitative analysis of the impedance data requires a more complicated, distributed equivalent circuit featuring CPE rather than pure capacitors. A *R-CPE* was chosen as a generalised one, which is practically used to empirically describe the OER [9], electrode–electrolyte interface resistance [10] and gas/liquid transport [11] in AEMWE. R_{HF}, R_{MF} and R_{LF} describe phenomenological charge transfer resistances assigned to high, middle and low frequencies, respectively.

The parameters of the stray impedance sub-circuit, which includes the parallel connection of an additional resistor and inductor *L-R* (Fig. S12A), can be estimated from the EIS measurements on the electrolyser cell in a short-circuit (SC) mode with a metal plate of thickness identical to the MEA thickness under potentiostatic control [12]. However, the response of stray impedance can be merged with the responses of ohmic resistance and fastest (HF) *R-CPE* element, which could disable the resolving of them. Therefore, we also performed the fitting of the separate high-frequency region of the spectra [13] to determine the values of the inductive element of stray impedance, for use in further full spectrum fitting.

Excluding the stray impedance and the ohmic resistance, only the HF and MF arcs could be identified in EIS spectra measured at 1.45 and 1.6 V (overpotentials of ~0.24 and ~0.4 V, respectively). For spectra obtained at a high cell voltage of 2.0 V (overpotential of ~0.8 V), some MEA configurations showed transport limitations (LF arc), with HF and MF arcs almost merged and inseparable in the fitting (Fig. S11E,F). In other cases, the pseudo-inductive feature

can be identified and evaluated (Fig. S11E,F). Both processes probably occur simultaneously, share the same low frequency range 0.1–1 Hz, and cannot be resolved with much credibility.

Finally, there are 9–12 equivalent circuit parameters, which makes it difficult to perform the fitting with reasonable confidence and low parameter variation. Hence, the following three assumptions were accepted in order to improve the fitting and decrease the result ambiguity.

1. The stray impedance sub-circuit was evaluated in the short-circuit measurements before the full cell testing. The obtained stray inductance value was used in further AEMWE impedance fittings as a fixed parameter.
2. The suggested high-frequency arc is related to the charge transport through the liquid electrolyte–electrode–interface [10]. The parameters of a high-frequency arc of AEMWE in operation are independent of cell voltage [14], which implies that this feature is not related to the kinetics of faradaic processes. It is well described with a R-CPE element with an α_{HF} coefficient close to 0.5. The fitting of spectra obtained at 1.45 V and 1.6 V using a fixed value of $\alpha_{HF} = 0.5$ enables reasonable evaluation of a HF and MF arc with ~100–1000-fold difference in their time constants for all tested AEMWE.
3. The summarised charge transfer resistance ($R_{ct} = R_{HF} + R_{MF}$) is considered.

The high accuracy of the fitting is proved by rather low values of the reduced chi-square factor of $5–50 \times 10^{-5}$. The parameters of fitting are presented in Tables S3–S7.

References:

[1] K.S.W. Sing, D.H. Everett, R.A.W. Haul, L. Moscou, R.A. Pierotti, J. Rouquerol, T. Siemieniewska, Reporting physisorption data for gas solid systems with special reference to the determination of surface area and porosity (Recommendation S 1984), Pure Appl. Chem., 57 (1985) 603-619.

[2] M. Schönleber, D. Klotz, E. Ivers-Tiffée, A Method for Improving the Robustness of linear Kramers-Kronig Validity Tests, Electrochim. Acta, 131 (2014) 20-27.

[3] N. Hensle, D. Brinker, S. Metz, T. Smolinka, A. Weber, On the role of inductive loops at low frequencies in PEM electrolysis, Electrochem. Commun., 155 (2023).

[4] Z. Qiu, Y. Ma, G.A. Niklasson, T. Edvinsson, An Electrochemical Impedance Study of Alkaline Water Splitting Using Fe Doped NiO Nanosheets, Physchem, 1 (2021) 69-81.

[5] I. Vincent, E.C. Lee, H.M. Kim, Comprehensive impedance investigation of low-cost anion exchange membrane electrolysis for large-scale hydrogen production, Scientific Reports, 11 (2021).

[6] A.T. Inc., Agilent Impedance Measurement Handbook, Agilent Technologies Inc., Santa Clara, CA, USA, 2009.
[7] G. Papakonstantinou, G. Algara-Siller, D. Teschner, T. Vidakovic-Koch, R. Schlögl, K. Sundmacher, Degradation study of a proton exchange membrane water electrolyzer under dynamic operation conditions, Applied Energy, 280 (2020).
[8] M.J. Jang, S.H. Yang, M.G. Park, J. Jeong, M.S. Cha, S.H. Shin, K.H. Lee, Z.Y. Bai, Z.W. Chen, J.Y. Lee, S.M. Choi, Efficient and Durable Anion Exchange Membrane Water Electrolysis for a Commercially Available Electrolyzer Stack using Alkaline Electrolyte, Acs Energy Letters, 7 (2022).
[9] S. Anantharaj, S. Noda, Appropriate Use of Electrochemical Impedance Spectroscopy in Water Splitting Electrocatalysis, Chemelectrochem, 7 (2020) 2297-2308.
[10] A. Khataee, A. Shirole, P. Jannasch, A. Krüger, A. Cornell, Anion exchange membrane water electrolysis using Aemion™ membranes and nickel electrodes, Journal of Materials Chemistry A, 10 (2022) 16061-16070.
[11] F. Razmjooei, T. Morawietz, E. Taghizadeh, E. Hadjixenophontos, L. Mues, M. Gerle, B.D. Wood, C. Harms, A.S. Gago, S.A. Ansar, K.A. Friedrich, Increasing the performance of an anion-exchange membrane electrolyzer operating in pure water with a nickel-based microporous layer, Joule, 5 (2021) 1776-1799.
[12] I.V. Pushkareva, M.A. Solovyev, S.I. Butrim, M.V. Kozlova, D.A. Simkin, A.S. Pushkarev, On the Operational Conditions' Effect on the Performance of an Anion Exchange Membrane Water Electrolyzer: Electrochemical Impedance Spectroscopy Study, Membranes, 13 (2023).
[13] T.P. Heins, N. Schlüter, U. Schröder, Electrode-Resolved Monitoring of the Ageing of Large-Scale Lithium-Ion Cells by using Electrochemical Impedance Spectroscopy, Chemelectrochem, 4 (2017) 2921-2927.
[14] A.S. Pushkarev, I.V. Pushkareva, S.P. du Preez, D.G. Bessarabov, PGM-Free Electrocatalytic Layer Characterization by Electrochemical Impedance Spectroscopy of an Anion Exchange Membrane Water Electrolyzer with Nafion Ionomer as the Bonding Agent, Catalysts, 13 (2023).

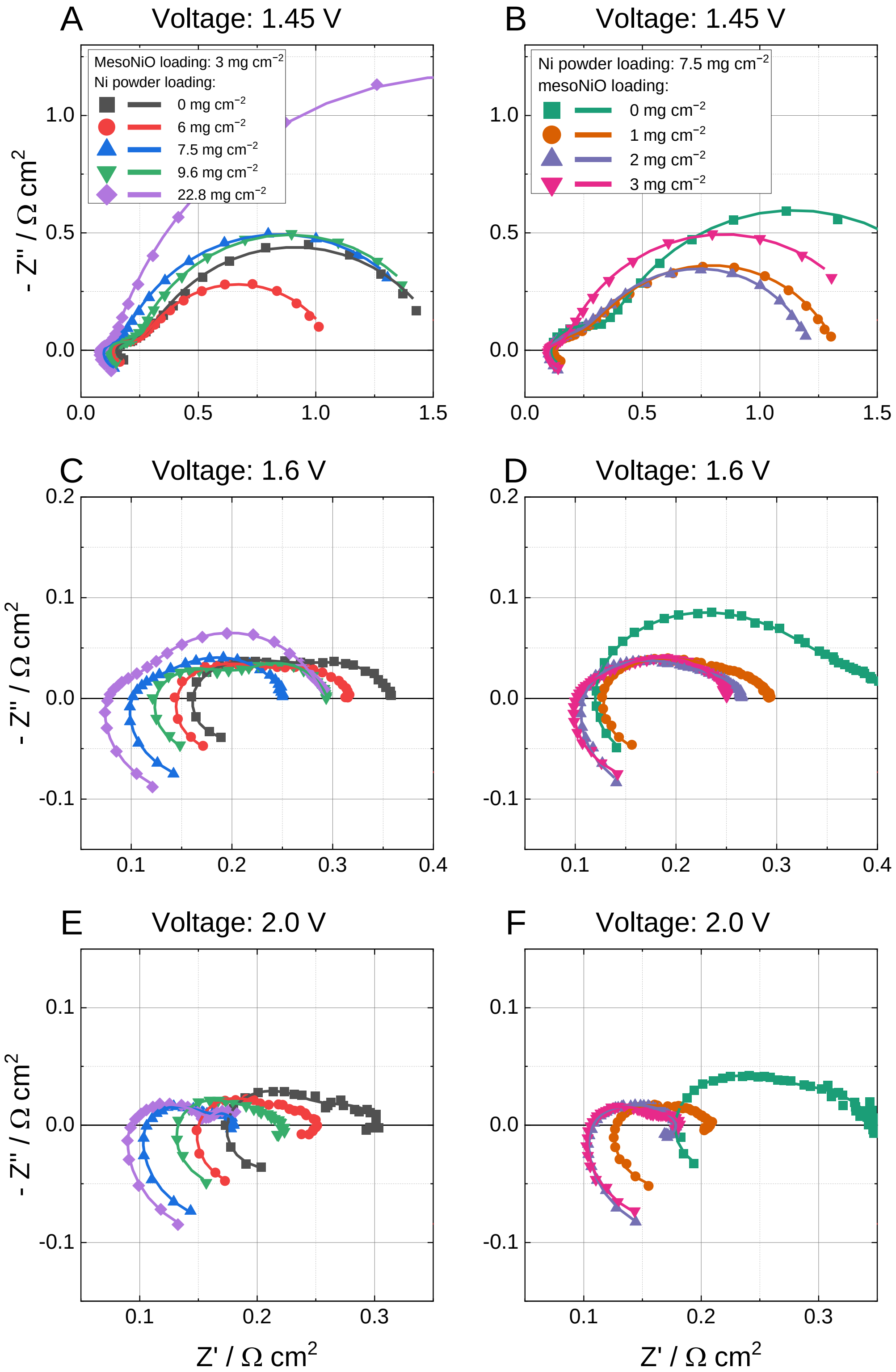

Figure S11. Measured Nyquist plots of AEMWE and the results of complex non-linear least-squares fitting according to the equivalent circuits (Fig. S12). Variation of nickel powder (A, C, E) and mesoNiO loading (B, D, F) are discplayed. Detailed fitting data are given in Tables S3–S7.

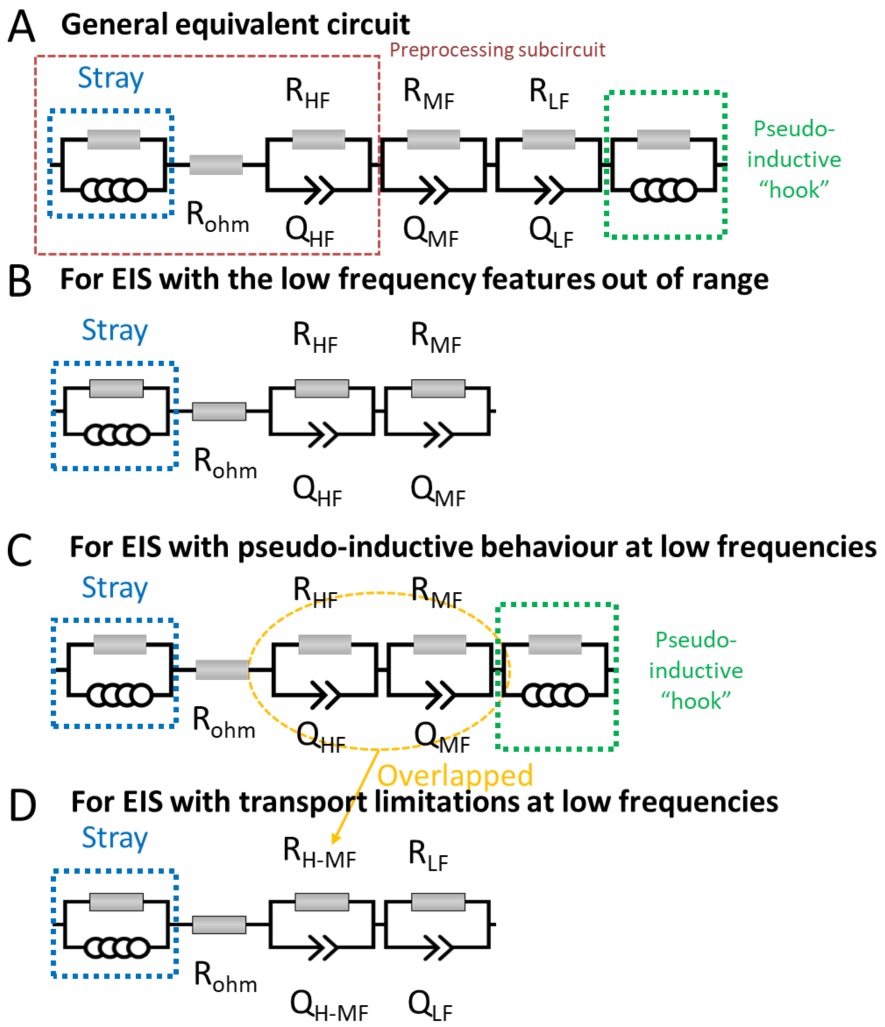

Figure S12. The general equivalent circuit (**A**) and the modified circuits for specific EIS data: with low frequency features out of the measured frequency range (**B**), with a pseudo-inductive feature (**C**), and with transport limitations in the low frequency range (**D**).

Table S3. The parameters of fitting for EIS obtained on AEMWE (Scheme 1H) at 1.45 V (equivalent circuit given at Fig. S11 **B**).

MEAs	Nickel powder loading, mg cm^{-2}	**0**	**6.0**	**7.5**	**9.6**	**22.8**	**7.5**	**7.5**	**7.5**
	mesoNi loading, mg cm^{-2}	**3**	**3**	**3**	**3**	**3**	**0**	**1**	**2**
Stray	R_S, Ω	0.0162	0.0204	0.0236	0.0195	0.0271	0.0221	0.0190	0.0296
	$I_S \cdot 10^{-7}$, H	1.14	1.14	1.32	1.04	1.41	1.19	1.16	1.37
R_{Ohmic}, Ω		0.0140	0.0139	0.0116	0.0107	0.0104	0.0087	0.0100	0.0100
HF arc	R_{HF}, Ω	0.0245	0.0247	0.0137	0.0225	0.0106	0.0384	0.0307	0.0340
	Q_{HF}, $Fs^{(\alpha-1)}$	0.5740	0.5006	1.9087	0.6034	2.2883	0.0530	0.5841	0.9173
	α_{HF}	0.5000	0.5000	0.5000	0.5000	0.5000	0.7206	0.5000	0.5000
MF arc	R_{MF}, Ω	0.1745	0.1125	0.1839	0.1791	0.4110	0.2198	0.1418	0.1245
	Q_{MF}, $Fs^{(\alpha-1)}$	1.8152	2.6213	2.8768	2.6336	2.7882	0.4295	0.9602	1.2466
	α_{MF}	0.7642	0.7557	0.8050	0.8189	0.8429	0.8104	0.7606	0.8039

Table S4. The parameters of fitting for EIS obtained on AEMWE (Scheme 1H) at 1.6 V (equivalent circuit given at Fig. S11 **B**).

MEAs	Nickel powder loading, mg cm^{-2}	**0**	**6.0**	**7.5**	**9.6**	**22.8**	**7.5**	**7.5**	**7.5**
	mesoNi loading, mg cm^{-2}	**3**	**3**	**3**	**3**	**3**	**0**	**1**	**2**
Stray	R_S, Ω	0.0163	0.0198	0.0240	0.0189	0.0277	0.0222	0.0193	0.0303
	$I_S \cdot 10^{-7}$, H	1.14	1.14	1.32	1.04	1.41	1.19	1.16	1.37
R_{Ohmic}, Ω		0.0144	0.0118	0.0100	0.0099	0.0077	0.0101	0.0099	0.0090
HF arc	R_{HF}, Ω	0.0256	0.0247	0.0089	0.0209	0.0088	0.0376	0.0282	0.0261
	Q_{HF}, $Fs^{(\alpha-1)}$	0.4965	0.4436	1.2982	0.4974	2.0718	0.0465	0.4835	0.7478
	α_{HF}	0.5000	0.5000	0.5000	0.5000	0.5000	0.7180	0.5000	0.5000
MF arc	R_{MF}, Ω	0.0095	0.0071	0.0160	0.0097	0.0236	0.0091	0.0024	0.0017
	Q_{MF}, $Fs^{(\alpha-1)}$	5.0919	4.4906	2.3844	3.5926	2.6192	5.2138	6.2804	11.6444
	α_{MF}	0.7487	0.8118	0.7033	0.8266	0.7845	0.6457	0.9492	0.9806

Table S5. The parameters of fitting for EIS obtained on AEMWE (Scheme 1H) at 2.0 V with pseudo-inductive feature manifested (equivalent circuit given at Fig. S11 **C**).

MEAs	Nickel powder loading, mg cm^{-2}	**0**	**6.0**	**7.5**	**9.6**	**22.8**	**7.5**	**7.5**	**7.5**
	mesoNi loading, mg cm^{-2}	**3**	**3**	**3**	**3**	**3**	**0**	**1**	**2**
Stray	R_S, Ω	0.0144	0.0187		0.0181			0.0181	0.0280
	$I_S \cdot 10^{-7}$, H	1.14	1.14		1.04			1.16	1.37
R_{Ohmic}, Ω		0.0146	0.0121		0.0099			0.0114	0.0086
HF arc	R_{HF}, Ω	0.0127	0.0134		0.0095			0.0082	0.0052
	Q_{HF}, Fs$^{(\alpha-1)}$	0.0717	0.1045		0.0783			0.0803	0.1714
	α_{HF}	0.7324	0.6720		0.7259			0.7297	0.7188
MF arc	R_{MF}, Ω	0.0148	0.0067		0.0086			0.0080	0.0084
	Q_{MF}, Fs$^{(\alpha-1)}$	15.947	6.660		6.408			4.924	3.707
	α_{MF}	0.3711	0.6164		0.4475			0.5485	0.5746
LF pseudo-inductive feature	R_{ind}, Ω	0.0038	0.0029		0.0031			0.0015	0.0028
	I_{ind}, H	0.0021	0.0032		0.0059			0.0010	0.0025

Table S6. The parameters of fitting for EIS obtained on AEMWE (Scheme 1H) at 2.0 V with pseudo-inductive feature manifested (equivalent circuit given at Fig. S11 **D**).

MEAs	Nickel powder loading, mg cm^{-2}	**0**	**6.0**	**7.5**	**9.6**	**22.8**	**7.5**	**7.5**	**7.5**
	mesoNi loading, mg cm^{-2}	**3**	**3**	**3**	**3**	**3**	**0**	**1**	**2**
Stray	R_S, Ω			0.0249		0.0279			
	$I_S \cdot 10^{-7}$, H			1.32		1.41			
R_{Ohmic}, Ω				0.0106		0.0095			
H-MF arc	$R_{H\text{-}MF}$, Ω			0.0130		0.0131			
	$Q_{H\text{-}MF}$, Fs$^{(\alpha-1)}$			2.4474		2.1339			
	α_{HF}			0.4406		0.4805			
LF arc	R_{MF}, Ω			0.0014		0.0029			
	Q_{MF}, Fs$^{(\alpha-1)}$			85.787		296.39			
	α_{MF}			1.0000		1.0000			

Table S7. The parameters of fitting for EIS obtained on AEMWE (Scheme 1H) at 2.0 V with pseudo-inductive feature manifested (equivalent circuit given at Fig. S11 **B**).

MEAs	Nickel powder loading, mg cm^{-2}	**0**	**6.0**	**7.5**	**9.6**	**22.8**	**7.5**	**7.5**	**7.5**
	mesoNi loading, mg cm^{-2}	**3**	**3**	**3**	**3**	**3**	**0**	**1**	**2**
Stray	R_S. Ω						0.0185		
	$I_S \cdot 10^{-7}$, H						0.0000		
R_{Ohmic}. Ω							0.0145		
HF arc	R_{HF}. Ω						0.0248		
	Q_{HF}. Fs$^{(\alpha-1)}$						0.0800		
	α_{HF}						0.6263		
MF arc	R_{MF}. Ω						0.0085		
	Q_{MF}. Fs$^{(\alpha-1)}$						2.0343		
	α_{MF}						0.6920		

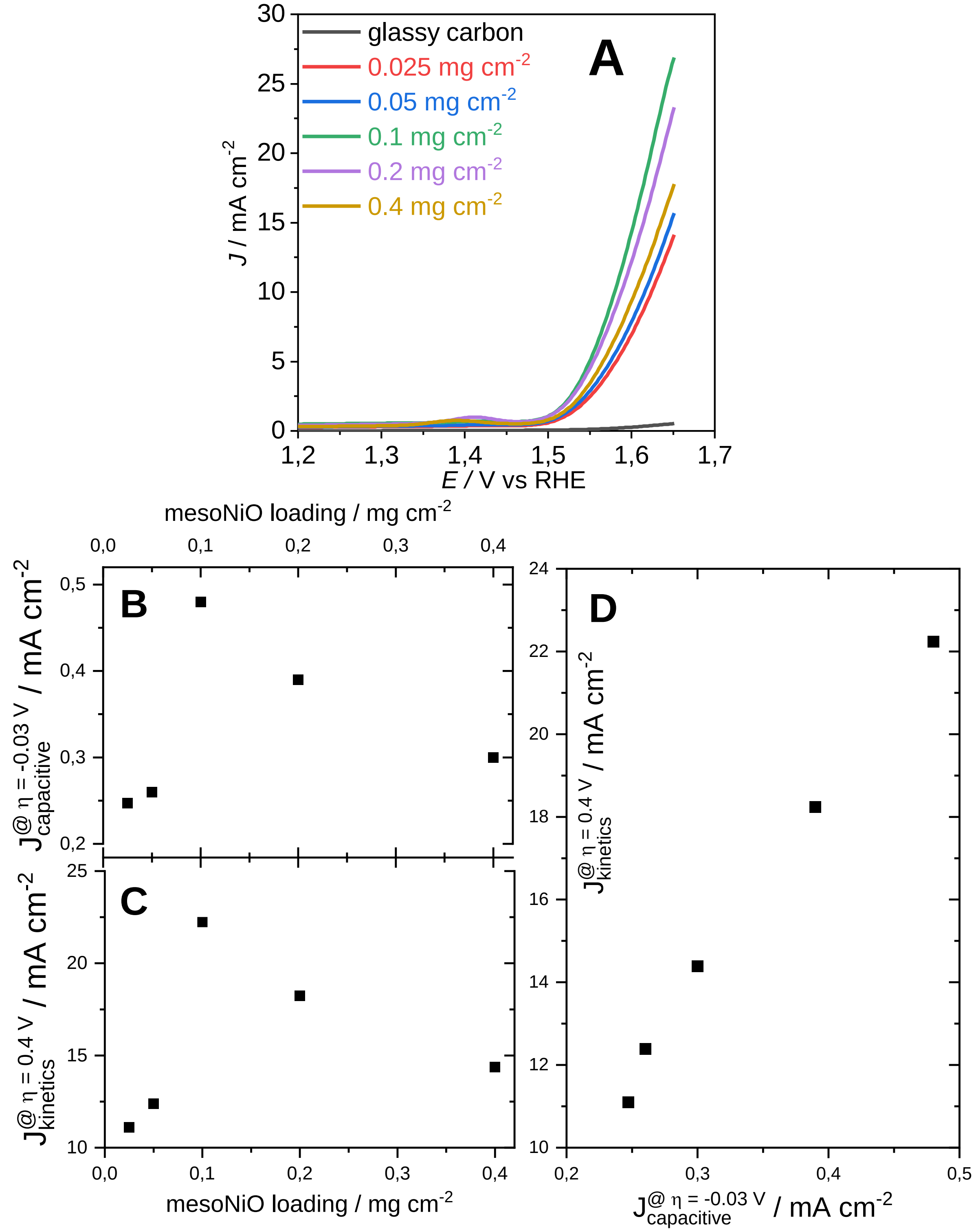

Figure S13. The effect of mesoNiO loading on the catalyst surface accessibility. **A:** linear sweep voltammograms were recorded in a three-electrode cell (Scheme 1B) on glassy carbon modified by different loadings of mesoNiO (scan rate 5 mV s^{-1}, 1 M KOH); **B** and **C:** the dependencies of capacitive currents and OER kinetic currents on the loading of mesoNiO; **D:** the dependence of OER kinetic currents on the capacitive currents on mesoNiO.

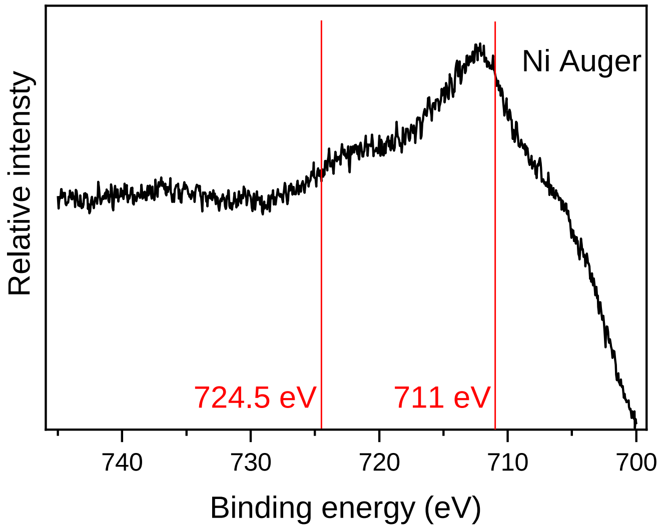

Figure S14. The absence of the Fe 2p peaks on XPS of postmortem AEMWE anode (9.6 mg cm^{-2} nickel powder loading, 3 mg cm^{-2} mesoNiO loading).

Table S8. The intensity of peak A and B of XAS spectra (Fig. 6C).

**Sample**	**Intensity of peaks**		
	Peak A	Peak B	Peak B/A
**NF (nickel foam)**	1.4600	0.1743	0.1194
**Ni powder**	1.5268	0.1946	0.1274
**Pristine Ni-on-NF**	1.5051	0.2137	0.1420
**Postmortem Ni-on-NF**	1.2003	0.3181	0.2650
**NiO powder**	1.2948	0.3750	0.2896
**Prinstine NiO-on-NF**	1.2818	0.3691	0.2879
**Postmortem NiO-on-NF**	1.2010	0.2962	0.2466

Table S9. The comparison of obtained performance with the relevant data for AEMWE and PEMWE (cell voltage at 1 and 2 A cm^{-2}).

Membrane Grade	Anode		Cathode		Feeding approach	MEA preparation approach[1]	Performance			Ref.
	catalyst (binder)	PTL type	catalyst (binder)	PTL type			Voltage, V @ 1 A cm^{-2}	Voltage, V @ 2 A cm^{-2}	T, °C	
Sustainion X37-50 (Dioxide Materials, US)	NiO (3 mg/cm^2) / Nafion binder	Ni foam	Pt/C (0.8 mg_{Pt}/cm^2) / Nafion binder	Ni foam	1 M KOH / 3 ml/min	CCS	1.74	1.92	60	This work
AEM water electrolysis										
PTFE-reinforced Sustainion X37-50 grade T	IrO_2 (2.0 mg/cm^2, Alfa Aesar, MA, USA) and 10 wt.% PTFE binder	Titanium felt (Bakaert, Belgium)	Pt/C (0.5 mg/cm^2, 46.6% wt.% of Pt) and 10 wt.% PTFE binder	carbon paper (Sigracet 39 BC, SGL carbon, Germany)	1M KOH, anode fed, 15–35 mL/min	CCS	1.8	2	60	[1]
PFTP-13[2]	IrO_2 (2.0 mg/cm^2, Alfa Aesar) and PFBP-14 binder	Titanium felt (Bakaert, Belgium)	Pt/C (0.5 mg/cm^2, 46.6% wt.% of Pt) and PFBP-14 binder	carbon paper (Sigracet 39 BC, SGL)	1M KOH, anode fed, 15–35 mL/min	CCS	1.69	1.78	60	[1]
Sustainion X37-50 grade T	IrOx (1.9 mg/cm^2) and Nafion binder	carbon paper (TGP-H-120, Toray)	Pt/C (1.2 mg/cm^2, 46.6% wt.% of Pt, TEC10E50E, TKK) and Nafion binder	carbon paper (TGP-H-120, Toray)	1M KOH	CCS	1.67	–	60	[2]

[1] CCM – catalyst coated membrane; CCS – catalyst coated substrate.
[2] PFTP-13 – poly(fluorenyl-co-terphenyl piperidinium-13); PFBP-14 – poly(fluorenyl-co-biphenyl piperidinium-14).

Sustainion X37-50 grade T	NiMo-NH_3/H_2 (3.0 mg/cm^2) and Nafion binder	Carbon paper	Fe-NiMoNH_3/H_2 (3.0 mg/cm^2) and Nafion binder	Carbon paper	1M KOH	CCS	1.63	–	60	[3]
PFTA-20[3]	IrO_2 (2 mg cm^{-2}) and PFTA-20 binder (5 wt.%)	Carbon paper (Toray TGP-H-060	Pt/C (1 mg cm^{-2}, 60 wt.% of Pt, HiSPEC 9100) and PFTA-20 binder (25 wt.%)	Carbon paper (Toray TGP-H-060	1M KOH	CCM	1.75	1.88	60	[4]
HTMA-DAPP[4] (50 µm wet thickness)	IrO_2 (0.75 mg Ir cm^{-2}, Premion) and HTMA-DAPP binder	Platinized Ti PTL	PtRu/C (0.36 mg Pt cm^{-2}HiSPEC 12100) and HTMA-DAPP binder	Carbon paper (29BC, SGL)	1M KOH	CCS	1.8	>2.05	60	[5]
PEM water electrolysis										
Nafion 115	IrO_2-based	Platinized Ti PTL (Bekaert 2GDL40–1,0)	Pt-based	Platinized Ti PTL (Bekaert 2GDL40–1,0)	DI water	CCM (Greenerity)	1.72	1.9	60	[6]

[3] PFTA-20 – poly(fluorenyl-co-p-terphenyl alkylene.
[4] HTMA-DAPP – hexamethyl trimethyl ammonium-functionalized Diels–Alder polyphenylene;

References:

[1] N. Chen et al., High-performance anion exchange membrane water electrolyzers with a current density of 7.68 A cm −2 and a durability of 1000 hours, Energy Environ. Sci. 14(12) (2021) 6338–6348.

[2] H. Koshikawa et al., Single Nanometer-Sized NiFe-Layered Double Hydroxides as Anode Catalyst in Anion Exchange Membrane Water Electrolysis Cell with Energy Conversion Efficiency of 74.7% at 1.0 A cm $^{-2}$, ACS Catal. 10(3) (2020) 1886–1893.

[3] P. Chen, X. Hu, High- Efficiency Anion Exchange Membrane Water Electrolysis Employing Non- Noble Metal Catalysts, Adv. Energy Mater. 10(39) (2020) 2002285.

[4] Y. Ma et al., Poly(fluorenyl alkylene)-based anion exchange membranes for high-performance water electrolysis, J. Chem. Eng. 480 (2024) 148225.

[5] J. Liu et al., Elucidating the Role of Hydroxide Electrolyte on Anion-Exchange-Membrane Water Electrolyzer Performance, J. Electrochem. Soc., 168 (2021) 054522.

[6] T. Lickert et al., Advances in benchmarking and round robin testing for PEM water electrolysis: Reference protocol and hardware, Appl. Energy, 352 (2023) 121898.
